# Understanding and addressing the obstacles to a trial of face masks in schools: a mixed-methods feasibility study

**DOI:** 10.1186/s40814-026-01825-7

**Published:** 2026-05-22

**Authors:** Ru Jia, Sophie Carlisle, Adam W. A. Geraghty, Sofia Strommer, Kieran Ayling, Jonathan Ball, Kirsty Bolton, Ben Cowling, Lucy Fairclough, Karen Jones, Wei Shen Lim, Paul Little, Tricia McKeever, Joanne Morling, Simon Royal, Paddy Tighe, Katherine Bradbury, Kavita Vedhara

**Affiliations:** 1 Nuffield Department of Primary Care Health Sciences, University of Oxford, Oxford, UK; 2 Health Innovation East Midlands, Nottingham University Hospitals NHS Trust, Nottingham, UK; 3 Primary Care Research Centre, Faculty of Medicine, University of Southampton, Southampton, UK; 4 MRC Lifecourse Epidemiology Centre, University of Southampton, Southampton, UK; 5 Faculty of Medicine and Health Sciences, University of Nottingham, Nottingham, UK; 6 School of Mathematical Sciences, University of Nottingham, Nottingham, UK; 7 School of Public Health, The University of Hong Kong, Hong Kong, China; 8 School of Education, University of Durham, Durham, UK; 9 Student Recruitment and Admissions, University of Durham, Durham, UK; 10 Department of Respiratory Medicine, Nottingham University Hospitals NHS Trust, Nottingham, UK; 11 National Institute for Health Research (NIHR) Nottingham Biomedical Research Centre, Nottingham, UK; 12 Cripps Health Centre, University of Nottingham Health Service, Nottingham, UK; 13 School of Health Sciences, University of Southampton, Southampton, UK; 14 School of Psychology, Cardiff University, Cardiff, UK; 15 School of Medicine, Keele University, Keele, UK

**Keywords:** Face masks, Schools, Young people, Feasibility trial, RCT

## Abstract

**Background:**

Face masks are a commonly used public health measure to control the spread of airborne infections, including in school-aged children. However, no trial of their effectiveness has been conducted in school settings to date. We undertook a mixed-method study to explore the feasibility of conducting such a trial.

**Methods:**

This study followed a multi-phased mixed-methods approach. We first conducted a secondary analysis of qualitative data (39 young people) and five Patient and Public Involvement focus groups (11 young people, three teachers, two parents), to understand the barriers and facilitators relating to engaging with a trial of face masks. Strategies to enhance recruitment and engagement were generated based on this qualitative work. These were refined further in additional PPI focus groups with young people (*n* = 7). We then conducted a 6-week two-arm parallel cluster-randomised feasibility trial in a local school. Two year groups were cluster-randomised into one of the two arms: (1) school rules group (SR, control), following school policy at the time on the use of face coverings; (2) face-mask group (FM, intervention), wearing standard lightweight surgical masks while in an indoor setting for the duration of the school day. Primary outcomes were recruitment and adherence to trial procedures: completion of surveys capturing frequency of mask-wearing, impact of mask-wearing, mood and compliance with the provision of weekly saliva samples for the detection of viral infections.

**Results:**

A total of 93 pupils (29% of approached) participated in the feasibility trial and were randomised (SR = 46, FM = 47). The number of intervention participants wearing face masks at all times while at school increased from 7% at baseline to an average of 28% over trial weeks 1–5. Completion rate of weekly surveys varied between 15 and 100%, while provision of weekly saliva samples varied between 15 and 38% over the trial period.

**Conclusion:**

Recruitment and adherence in the trial were lower than those observed in trials conducted in other community or healthcare settings. Our data suggest that incentivising participants, giving participants greater agency in how to fulfil trial procedures such as giving them choices on where to collect saliva samples, and allowing survey completion during timetabled activities would be important considerations for a future trial.

**Supplementary Information:**

The online version contains supplementary material available at 10.1186/s40814-026-01825-7.

## Key messages regarding feasibility

- *What were the uncertainties regarding feasibility?* The key uncertainties related to the feasibility of conducting a randomised trial on the effectiveness of face masks in school settings were the ability to recruit and retain school-aged participants and to collect the required data. In particular, we were uncertain about pupils’ adherence to trial procedures, including consistent mask-wearing, the completion of weekly surveys, and the provision of saliva samples for analysis.
- *What are the key feasibility findings?* We found that recruitment and adherence rates among school-aged children were lower than those typically observed in other community or healthcare settings. Adherence to mask-wearing and biological sample collection presented particular challenges. However, we identified several factors that facilitated participation, which may be leveraged to improve future trial implementation.
- *What are the implications of the feasibility findings for the design of the main study?* While the study confirmed that a trial on the effectiveness and impact of face masks in schools is feasible, it also highlighted unique challenges related to recruitment and adherence in this setting. The findings suggest that future trials should incorporate strategies to address these challenges, such as providing incentives for participation, offering pupils greater agency and involvement in trial processes such as giving them choices on where to provide saliva samples and on the types of face masks they could wear, and adapting data collection procedures to better fit the school schedules.

## Background

### Face coverings and infection control

The burden inflicted by respiratory infections is considerable for both primary and secondary care [1], with the associated pressures intensifying significantly when these conditions surge in the winter months [2]. A systematic review of the costs of acute respiratory infections (ARIs) in older adults between 2001 and 2021 revealed that the average inpatient costs, prior to the Coronavirus 2019 (COVID-19) pandemic, were €166 in South-East Asia, €1188 in Europe, and €17834 in North America per patient per episode [3]. In the UK, analysis of the direct medical costs of such infections for the NHS between 2001 and 2009 suggests that they cost £86 million per annum [4]. The emergence of COVID-19 intensified this burden and, in many countries, stretched healthcare services to breaking point. This necessitated the introduction, globally, of a raft of public health measures designed to control the spread of the infection. Chief amongst these was face coverings. Early in the course of the COVID-19 pandemic, the public health advice on face coverings was fairly consistent, with many countries advocating their use only in people with symptomatic infection and in healthcare settings [5]. However, countries then diverged significantly in their advice for the public, in part to constrain the demand for face coverings, but also because the evidence for effectiveness in otherwise healthy people was limited [6]. Two main issues changed the narrative on face coverings. First, many argued that the devastating consequences of the pandemic warranted a return to the precautionary principle, i.e. that face coverings should be encouraged on the basis that their potential for benefit, far outweighed their potential for harm [7–9]. Second, the evidence in support of aerosol transmission of SARS-CoV-2 started to grow [7, 10], leading eventually to recognition by the World Health Organization (WHO) and others that the virus is spread through the air (involving both droplets and aerosols [11].

### The contested evidence on face coverings

While both considerations fuelled support for mask wearing, an updated Cochrane review of the trial evidence in 2020 concluded “The pooled results of randomised trials did not show a clear reduction in respiratory viral infection with the use of medical/surgical masks during seasonal influenza” and served to increase uncertainty and confusion regarding their effectiveness. A recent update of this review appears not to have settled the debate. The review now includes 15 trials (14 trials included in the previous iteration of this review) focusing on using masks and a combined sample size of 14,221. However, the conclusions remain largely the same (i.e. “There is uncertainty about the effects of face masks.”) and many of the limitations with the evidence remain [12].

One of biggest limitation of current evidence on face coverings is poor adherence. As the Cochrane review (see Jefferson et al. [12]) pointed out, the majority of current trial evidence on face covering did not report, or reported poor adherence (e.g. 25–47% adherence as reported in [13, 14]. A second and related issue is that adverse effects of face coverings were also poorly reported or not reported at all. Discomfort such as warmth, respiratory difficulties, and other general discomfort were the most frequently reported adverse effects, especially among children, which may contribute to reduced adherence. A third limitation is the poor generalizability of evidence from contexts such as hospital or household settings (89%) to community settings due to a lack of trials in these settings. Indeed, one important context in which no trial evidence currently exists is schools.

### Why schools are critical for infection spread

Schools play an important role in the transmission of infections, especially respiratory infections due to the high frequency and proximity of contacts in often poorly ventilated environments [15, 16]. A study in 2008 suggested that school-aged children (10–19 years) had the highest number of daily contacts, compared to other age groups, which was associated with the highest incidence of respiratory infections in that age group [16]. Infections in schools may also contribute to “spillover” infections (i.e. infections that extend into households and the wider community). Evidence from the 2009 H1N1 pandemic showed that the opening of schools was correlated with the onset of community transmission of H1N1 in the USA [17, 18]. Other data also suggest that school closures during holiday periods prevent 14–17% of seasonal influenza cases in adults and 18–21% in children [15]. There is, therefore, a need to consider and evaluate interventions that seek to reduce the risk of respiratory infections in schools, including face coverings.

However, as highlighted by Dawson et al. [19], conducting such a trial can be challenging due to the relative inexperience of schools in participating in research. Accordingly, it is recommended that school-based trials should be co-created with schools [20, 21]. Here we describe the co-creation activities we undertook to inform the design of a feasibility cluster randomised controlled trial and the findings of this trial. Our objectives were:

1. To understand the barriers and facilitators to engagement with a future trial, with a particular focus on developing strategies to optimise trial recruitment and adherence with trial procedures, including mask wearing.
2. To design and undertake a feasibility trial to examine the feasibility and performance our approach, as measured by recruitment and adherence to trial procedures.

## Methods and results

### Understanding the barriers and facilitators to engagement with a future trial

**Fig. 1:**
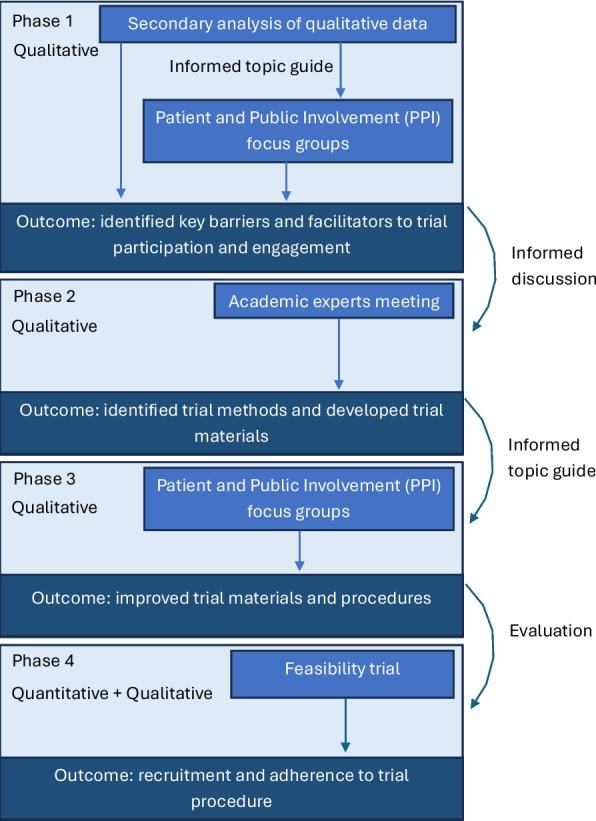
Procedures involved in understanding the barriers and facilitators to engagement with a future trial

We undertook three main activities, following a multi-phased approach, to understand the barriers and facilitators to engagement with a future trial. These three activities included analysis of existing qualitative data, Patient and Public Involvement (PPI) focus groups with young people, teachers, and parents, and pre-feasibility-trial PPI focus groups with young people only. Figure 1 summarises these activities.

#### Analysis of existing qualitative data

Secondary analysis of existing qualitative data from the Teenagers’ experiences of the COVID-19 (TeC-19) Study was conducted. Sixteen focus groups with a total of 39 young people (22 male and 17 female) between the age of 15–18 years were included in the secondary analysis. The TeC-19 study explored young people’s experiences of the COVID-19 pandemic (full details available: Strömmer et al. [22]). In brief, online focus group discussions were conducted over the first 12 months of the pandemic. Participants were young people from the community in or near the English cities of Southampton, Winchester, Manchester, Brighton, and Birmingham. Information relating to young people’s experiences of and views on face masks/coverings were available in the TeC-19 study data. We conducted a secondary analysis of these data to identify barriers and facilitators to the use of face masks/coverings.

All transcripts were imported into NVIVO. To identify relevant discussion, transcripts were searched using NVIVO’s inbuilt text searching function. Transcripts were searched for words and phrases including: ‘mask’, ‘covering’, ‘face mask’, ‘wear’, including both exact matches and stemmed words. We identified any discussion relating to face masks and their use in any context, including schools. Consistent with Ntoumanis et al. [23], we considered that information on how young people view themselves and their motivation could be helpful in illuminating strategies to promote engagement in the trial and trial procedures [23]. Thus, we also identified discussions relating to ‘identity’, i.e. how young people viewed themselves in the pandemic. All transcripts were read in full to identify such discussion as this was more difficult to carry out as a text search.

Relevant sections of transcripts that were identified either through NVIVO searching or full text reading were then analysed following Braun and Clarke’s 2006 inductive thematic analysis approach (REF) [24]. First, the researcher (SC) familiarised themselves with the relevant data by reading and re-reading the identified sections and surrounding parts of the transcripts to ensure understanding of the context. Then the sections were coded in terms of unit of meaning. Some text had more than one code as it was related to multiple ideas, experiences or beliefs. Any text unrelated to face masks or identity was not coded. Once initial codes were generated, they were grouped into themes and sub-themes (see Supplementary Appendix Table A1). This was an iterative process in which codes and sub-themes were reviewed and renamed as the themes and subthemes developed. These themes and sub-themes informed both the development of the topic guide for the following PPI focus groups and the design of the feasibility trial (see “Identifying trial methods” section).

#### Patient and public involvement focus groups

We next established PPI focus groups specifically to (1) understand young people’s, teachers’ and parents’ views on face masks in schools; (2) identify key barriers and facilitators to participating and engaging in a feasibility trial of face masks; and (3) understand views on the proposed trial design (e.g. length of observation period, frequency of data collection). Information gathered from these PPI focus groups informed the design of the feasibility trial.

To be consistent with the year groups to be targeted in the feasibility trial, members of PPI focus groups were either young people aged 16–18 years old who were in full-time education, parents of people aged 16–18 years old or teachers of 16–18-year-olds. We focused on this age range so that young people would be able to consent for themselves, rather than requiring the additional involvement of parents to provide informed consent.

Members were primarily recruited through a social media campaign involving but not limited to Facebook, Twitter, and Instagram. In addition, snowball recruitment [25] was also adopted where people who expressed interest in the study were asked to invite others who might be eligible. Recruitment of the PPI focus groups took place from 2nd December 2021 to 18th January 2022. Recruitment of young people, teachers, and parents was conducted independently from each other.

Potential members completed a registration form via JISC Online Survey to indicate whether they were young people, teachers, or parents, and their availability. Gender and attitudes towards masks were also collected through the registration form by the item ‘Would you describe yourself as…’ ‘pro-masks/anti-masks/not bothered either way’ as options. This was to recruit PPI focus group members of a wide range of views and across genders. They were also asked to indicate reasons for not wearing a mask from a list (see Supplementary Appendix B). Young people and teachers were also asked if they wore masks in classrooms. Selection of time and members for focus groups was based on the number of people selecting the same time slot(s) provided. Members who were invited to the focus group received a link to the focus group meeting on Microsoft Teams via email. All members received a £10 voucher as compensation for their participation.

**Fig. 2:**
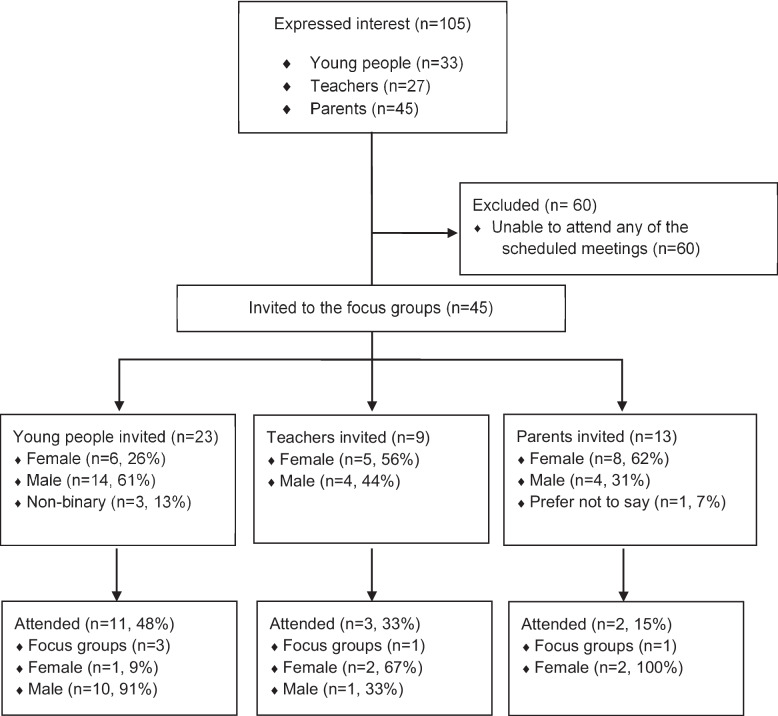
Recruitment procedure of the PPI focus groups

The recruitment procedure of the focus groups is shown in Fig. 2. In short, 33 young people, 27 teachers and 45 parents expressed interest and were invited to take part in the focus groups. A total of 11 young people, three teachers, and two parents attended the focus groups. The focus groups took place between 21 st December 2021 and 22nd January 2022.

Topic guides for the PPI discussion groups were developed iteratively based on the secondary analysis reported in 2.1.1. Included topics and example questions are shown in Supplementary Appendix B.

All focus groups were recorded and transcribed verbatim. As the purpose of these groups was to inform our primary research methods, our approach to analysis was to undertake a basic content analysis in which transcripts were analysed systematically and described descriptively to identify themes as described by Drisco and Maschi [26]. All transcripts were read through, and quotations relevant to the main topics (i.e. barriers and facilitators to trial participation and engagement with trial procedures) were extracted and grouped into categories, which were pre-identified key target behaviours for the following trial.

For category ‘trial participation’, 16 facilitators and 9 barriers were identified. For ‘engagement with trial procedures’, 14 facilitators and 13 barriers were identified. Summary of the barriers and facilitators identified in the focus groups is presented in Table 1.

**Table 1:** Summary of barriers and facilitators identified in the secondary analysis and PPI focus group and proposed solutions and associated theoretical component

1	Barriers and facilitators identified	Methodology/Intervention components	Associated theory (or design issue where indicated)	Proposed solution
2	**Target behaviour: recruitment into the RCT**			
3	Barrier: Fear of COVID-19 has reduced	Intervention components	Protection motivation theory [27]	The trial would not focus on COVID-19, but future risk
4	Barrier: feeling of being forced to take part in the trial Facilitator: understanding the importance of the trial Facilitator: communicate the research with students and parents clearly and transparently, making sure they understand the research and had the opportunity to ask questions before taking part	Intervention components	Self-determination theory [28]	The research team would communicate the research with students and parents clearly and transparently, making sure that they understood the rationale for taking part in the research (i.e. the benefits of participation) so that they can make up their own mind about whether to take part, and had the opportunity to ask questions before taking part (Letters and Live Q&A)
5	Barrier: parental consent viewed as important for participation	Methodology	Of relevance to recruitment strategy	Ideal population would be pupils older than 16 (so able to consent themselves) Introductory information (letters, meetings) would be provided prior to inviting pupils to consent to the project
6	Barrier: inappropriate timing of the trial (during summer when wearing masks is more difficult or during exam season when pupils don’t have time) or inappropriate groups of participants who might not be willing to take part	Methodology	Of relevance to recruitment strategy	Ideal population would be: (a) not an exam year; (b) but also at school full-time. This is to ensure that mask wearing coincides with where their greatest risk of exposure is likely to be Ideal year group: year 12 and year 13
7	Barrier: unclear about what the study involves and risks of taking part	Intervention components	Social cognitive theory and self-determination theory [28, 29]	The research team would communicate the downsides of participating in the research with students and parents clearly and transparently, making sure they understand the research and had the opportunity to ask questions so that they can make an informed choice of whether to take part (Letters and Q&A sessions with pupils and parents)
8	Barrier: trial stage being too short or too long	Methodology	Of relevance to trial design	Key feature of trial: Duration of mask wearing: 6 weeks
9	Facilitator: rules around mask wearing are easy to remember and follow	Intervention components	Social cognitive theory [29]	Key features of trial: a) The instructions for mask-wearing would be made clear and easy to remember: i) pupils in the face mask (intervention) group would be asked to wear a face mask when indoors at school, apart from eating and drinking or if they had medical exemptions ii) pupils in the school rules (control) group would be asked to follow the school’s guidance on face masks at the time during the trial b) Posters on how to wear masks would be developed, with PPI members
10	Facilitator/study design: supply or new/fresh masks	Intervention components	Social cognitive theory [29]	Key features of trial: a) Fresh masks would be supplied to all pupils and teachers twice daily b) Choice of masks: surgical masks c) Replacement masks to be provided to enable pupils to change masks after each break in the day
11	Barrier: high frequency of saliva samples	Intervention components	Social cognitive theory [29]	Key feature of trial: Saliva sample would be provided by pupils once a week for duration of trial
12	Study design: students to report mask wearing behaviours/adherence	Methodology	Of relevance to trial design	Key features of trial: a) Self-report of mask wearing by both pupils (weekly) and teachers Teachers: Estimated percentage of students wearing masks during each class
13	Barrier: teachers unable to report/monitor mask wearing behaviours/adherence (Staff time and availability)	Intervention components	Social cognitive theory and self-determination theory [28, 29]	Key features of trial: See 12 The intervention would minimise pressure on staff by: a) Making it easy to remember procedure by only conducting the trial within 1 year group b) Making completion of data collection (e.g. mask wearing) extremely simple c) Going into schools to talk in assembly to support staff to talk about the study d) Providing the school with incentives to cover staff time spent on the study
14	Facilitator: wanting to know whether masks work. Young people would like to know whether masks work and are they necessary instead of simply doing what they have been told	Intervention components	Self-determination theory [28]	The rationale for the study for pupils should include the social justice considerations – things should be fair (e.g. if we were to ask pupils to wear masks to protect others, we should know if they work and how well they work)
15	Facilitator: consider needs of schools and young people	Intervention components	Self-determination theory [28]	Emphasis in the rationale for the study that schools and young people have not been well-served in the pandemic which means we did not have a strong evidence base of what was effective in protecting young people
16	Facilitator: enjoyment of being part of research	Intervention components	Self-determination theory [28]	Emphasise in the rationale for the study that the study is an opportunity to engage with research and scientists to both young people and schools
17	Facilitator: being able to contribute to the society	Intervention components	Self-determination theory [28]	Emphasise in the rationale for the study that it is an opportunity for young people to influence the science affecting them
18	Facilitator: being in touch with higher education	Intervention components	Self-determination theory [28]	This study would be promoted as an opportunity to engage with research and scientists to both young people and schools
19	Facilitator: having 1 year group. A certain number of staff might already be overseeing the pupils	Intervention components	Social cognitive theory and self-determination theory [28, 29]	See 13
20	Facilitator: approach head teachers and leadership group to advertise the study	Methodology	Of relevance to trial design	Before the trial starts, the research group would have informal discussions with head teacher and school leadership group
21	**Target behaviour: mask wearing**			
22	Barrier: lack of information of the rationale for wearing face masks	Intervention components	Self-determination theory [28]	The research team would explain the rationale for engagement with the trial procedures in a way that is meaningful to young people, as young people are especially sensitive to external pressure
23	Barrier: young people feel society has not prioritised them and has in some cases blamed or scapegoated them for aspects of the pandemic. This could create an unwillingness to adhere to societal norms		Self-determination theory [28]	The rationale for the study would draw on a social justice arguments to show that young peoples’ and schools’ needs should be considered The research team would explain the rationale for engagement with the trial procedures in a way that is meaningful to young people, as young people are especially sensitive to external pressure
24	Barrier: social context	Intervention components	Self-determination theory [28]	See 14
25	Barrier: discomfort	Intervention components	Social cognitive theory and self-determination theory [28, 29]	This study would acknowledge the difficulties of wearing masks (i.e. showing understanding of the issues). Suggestions would be given to resolve these issues (e.g. pupils would be allowed to have short breaks from wearing a face mask if they experience discomfort) The recruitment would focus on the positive side of the trial Key features of trial: See 9 and 10
26	Barrier: inconvenience	Intervention components	Social cognitive theory and self-determination theory [28, 29]	See 25
27	Barrier: forgetting	Intervention components	Social cognitive theory [29]	See 10
28	Facilitator: teachers wearing masks (solidarity)	Intervention components	Self-determination theory [28]	Key feature of trial: Teachers of face mask (intervention) group would be asked to wear face masks when teaching this year group
29	Facilitator: peer influences	Intervention components	Self-determination theory and Theory of Planned Behaviour [28, 30]	Although the trial would only involve 2-year groups, the whole school would be made aware of the research to build peer support for the year groups involved
30	Facilitator: having reminders	Intervention components	Social cognitive theory [29]	Key features of trial: a) Poster reminders would be put up in the school b) See 10
31	Facilitator: fitting the study into existing school activities/making the behaviour easier	Intervention components	Social cognitive theory [29]	Key features of trial: The trial would be built into the weekly personal development session at school
32	Facilitator: getting feedback	Intervention components	Social cognitive theory [29]	Key feature of trial: Feedback on the trial progress in the previous week (e.g. the number of pupils taking part, the percentage of pupils wearing face masks, self-reported mood) would be summarised and reported to the pupils and teachers during the weekly research visit
33	Facilitator: incentives	Intervention components	Social cognitive theory [29]	Key feature of trial: After discussion with school about incentives, it was decided that: a) Pupils would not be incentivised individually for taking part in the trial; b) A certificate for participation will be given to the school at the end of the trial; c) Teachers who participated in the study would be incentivised to reimburse staff time spent on the study
34	**Target behaviour: providing saliva samples**			
35	Barrier: in front of other people		Of relevance to trial design	Key feature of trial: Saliva samples would be collected at school during the weekly research visit
36	Barrier: unclear what the samples are for and how to collect the samples	Intervention components	Social cognitive theory [29]	The research team would state clearly in the letters to parent and pupils, information sheet, and consent form that the collection of saliva samples for this feasibility study only aims to test the feasibility of weekly collection of saliva samples in schools. The saliva samples collected would only be counted and then discarded, and would not be analysed This will also be addressed at any meeting or Q&A sessions with parents and pupils
37	Barrier: too frequent	Intervention components	Social cognitive theory [29]	Key features of trial: a) Saliva samples would be collected weekly for 6 weeks b) See 35
38	Facilitator: school having a system in place/Done at school	Methodology	Social cognitive theory [29]	Key features of trial: The weekly research visit would take place at the personal development session every Tuesday morning. Pupils from the selected year groups would be asked to attend. For those who took part in the study, they would be asked to complete a self-report survey, and provide saliva samples during this session (data collection). One expert from the research team would be invited to interact with the pupils after the data collection, at the research visit
39	Facilitator: having reminders	Intervention components	Social cognitive theory [29]	Key features of trial: a) Poster showing how to wear face masks appropriately and how to provide a sample would be put up in the school Have posters at school as reminders b) During each weekly research visit, pupils will be reminded to continue wearing face masks and providing saliva samples
40	Facilitator: having more information/instructions on how to take the saliva samples	Intervention components	Social cognitive theory [29]	Key features of trial: a) Clear and simply instructions on how to provide a saliva sample would be provided to pupils in written format and explained verbally b) Posters showing how to provide a saliva sample would be developed, with PPI members

#### Identifying trial methods

Following the identification of the key barriers and facilitators through the qualitative work, we sought to identify strategies to address these. This entailed a virtual group meeting with academic experts from the fields of public health, health psychology, and education. Barriers and facilitators were mapped onto theories where possible and theory-informed strategies were developed to address barriers and harness facilitators. Summary of the results is presented in Table 1. For details, see Supplementary Appendix Table B1.

### Pre-feasibility trial PPI

The resulting trial methods and the approach to recruitment and maximising adherence to trial procedures were explored in a further three pre-trial PPI groups of n = 7 young people in total (15–18 years, three female, four male). Feedback was sought on all participant-facing trial materials (i.e. the study introduction letter, participant information sheet, and all trial materials) and an oral presentation designed to launch the trial. Seven PPI members provided written or oral feedback on study materials and five provided feedback on the presentation. These PPI meetings were held online via Microsoft Teams. All PPI members received a £10 voucher as compensation for their participation.

### The feasibility trial

#### Aims and objectives

The feasibility trial was conducted to examine the effectiveness of our approach, as measured by recruitment and adherence to trial procedures. Specifically, our objectives were:

1. To examine the rate of participation and reasons for declining participation;
2. To examine adherence to trial procedures, including completion rates of daily surveys, self-reported mask-wearing behaviours, and provision of saliva samples;
3. To explore the self-reported impact of mask-wearing on health and well-being among pupils.

#### Design and randomisation

A two-arm, parallel, cluster randomised controlled feasibility trial (RCT) limited to two clusters in a single school was conducted for six weeks from 13/09/2022 to 18/10/2022. Clusters were randomised at the year group level to reduce the risk of contamination between groups: (1) face mask group (FM, intervention condition); and (2) school rules group (SR, control condition). Group allocation was determined and contained in a sealed envelope by staff members who were not part of the trial team. All data were summarised descriptively. Protocol of the feasibility trial was pre-registered at Open Science Framework (10.17605/OSF.IO/G83CN). We did not conduct a sample size calculation for the feasibility trial.

#### Blinding

The research team and participants were blind to group allocation until recruitment into the trial and informed consent had been obtained. However, due to the nature of the intervention, it was not possible to blind participants or the research team to group allocation post-randomisation.

#### Ethical approval

Ethical approval was obtained from the University of Nottingham Faculty of Medicine and Health Sciences Research Ethics Committee (reference number FMHS 30–0722).

#### Setting, recruitment and participants

The trial took place in a single secondary school (Nottinghamshire, UK) and all pupils in years 12 and 13 (aged 16 years and older), and staff teaching these year groups were invited to participate.

Recruitment of teachers: PPI feedback revealed that pupils would feel supported in wearing masks if their teachers also agreed to do so. Thus, we sought to encourage mask wearing by teachers when teaching pupils in the intervention year group. Recruitment and informed consent of teachers was conducted before the start of the academic term and return of pupils, during a continuous professional development (CPD) day. Prior to the CPD day, an introductory letter outlining the study was sent to all staff from the head teacher. On the CPD day, teachers attended a presentation on the study were provided an opportunity to raise questions and concerns and then invited to consent to participate. Inclusion criteria for teachers were limited to (1) teaching year 12 or 13 at the participating school and (2) being able to give informed consent. Thirty-three teachers agreed to take part in the feasibility trial.

Recruitment of pupils: in advance of pupil recruitment, study information letters were sent to all year 12 and 13 pupils, as well as separate letters to their parents from the Head Teacher. This was followed by a formal presentation by the study team to year 12 and 13 pupils. The rationale for the study and what it would involve was presented, followed by a question-and-answer session. Pupils were encouraged to either raise their hands or scan a QR code and submit questions anonymously. *N *= 321 pupils were present at the meeting and following the presentation all eligible pupils were invited to participate. Inclusion criteria for pupils were (1) studying at the participating secondary school and being in year groups 12–13 (aged at least 16 years), (2) being able to give informed consent, and (3) being able to read English. Pupils indicated their willingness to participate by scanning another QR code which led to an online consent form on which they expressed whether or not they wished to take part in the trial. A total of 93 (29%) agreed to participate. The results of the randomization were revealed after pupils had consented, with year 13 pupils allocated to the face mask condition (*N *= 47, 53% female) and year 12 pupils (*N *= 46, 43% female) allocated to the control condition.

#### Study conditions

*Intervention group (FM).* Participants in FM were asked to wear a standard lightweight surgical mask conforming to EN 14683 standard while in an indoor setting for the duration of the school day over 6 weeks. Teachers were also asked to wear these masks while teaching the intervention group. Masks were provided free to all participants with sufficient face masks made available to enable at least 2 fresh masks to be worn per day. This was to ensure that the cost and availability of face masks were not a barrier to their use. Guidance on the appropriate way to wear a face mask was also provided (for example, see Supplementary Appendix C). Participants were not required to wear a mask when outdoors on school premises, when eating or drinking, if they were medically exempt from wearing a mask, or when not on school premises.

*Control group (SR).* Participants in SR were asked to follow school policy at the time on the use of face coverings over the trial period. For the duration of the trial the school’s guidance was that masks could be worn at the discretion of the pupil.

#### Trial procedure

Over the 6-week observation, participants in both arms of the trial were asked to engage in additional trial activities. Details of these trial activities are described in Sections 2.2.6–2.2.8. These took place once a week and in person at the school under the supervision of the research team and teaching staff. The sessions were scheduled into the timetable to coincide with a lesson (whole-year assembly) when most pupils from the participating year groups were expected to be present. However, as the observation period progressed it became clear that attendance at the assembly was not mandatory and attendance declined. Accordingly, by week 3, data collection was moved to tutor groups. Details of how the data collection procedure was adjusted during follow up are described below and in Supplementary Appendix C.

#### Weekly survey

Participants in both groups completed an online survey weekly. Survey items included the following measures:

*Self-reported adherence with mask-wearing.* This was measured by a single item ‘How often over the past week did you wear a face covering while indoors at schools?’. Participants were asked to report on the following scale: never (0%), Up to 25% of the time, between 25 and 50% of the time, between 50 and 75% of the time, between 75 and 100% of the time, and always (100%). A ‘Not applicable’ option was given for participants who were medically exempt from wearing a face mask.

*Impact of mask-wearing on learning, interactions with people, physical health, and mental health*. These outcomes were measured using 6 questions in the format of ‘To what extent has wearing a face covering affected your learning/interactions with your friends/interactions with your teachers/interactions with other people in general/your physical health/your mental health over the past week?’. Participants rated on a five-point Likert scale (1 = extremely negatively—3 = not much/same as not wearing a face covering—5 = extremely positively).

*Mood.* Positive and negative mood were measured by the Scale of Positive and Negative Experience [31], with a total score between 6 and 30 for positive mood and 6–30 for negative mood, and higher scores indicating stronger positive or negative mood.

*Loneliness.* Loneliness was measured by an adjusted version of the Three-Item Loneliness Scale [32], with a total score between 3 and 9 and higher scores indicating greater levels of loneliness.

For details, see Supplementary Appendix C. In the last weekly survey, pupils were also asked to provide feedback on the trial and methods employed, including providing suggestions to improve recruitment and adherence to trial procedures.

Teachers also completed a weekly survey in which they estimated the proportion of mask-wearing for each class they taught that was randomised to FM only.

#### Saliva samples

In a future trial we anticipated determining mask effectiveness by measuring the presence/absence of common respiratory viral infections in saliva. Thus, pupils were asked to provide a saliva sample once a week, although in the present study these were not to detect infection, but rather to assess willingness to provide samples. Thus, we only recorded the sample return rate each week. No samples were analysed for the presence of infection. Sample collection was non-invasive involving drooling or spitting into a tube via a straw [33]. From baseline to week 2, samples were collected during the scheduled assembly. From week 3, this moved to tutor groups (see Supplementary Appendix C). In seeking to optimise sample return rates, pupils received small incentives (confectionery) for providing samples from weeks 4–6.

## Results

### Participant characteristics

**Fig. 3:**
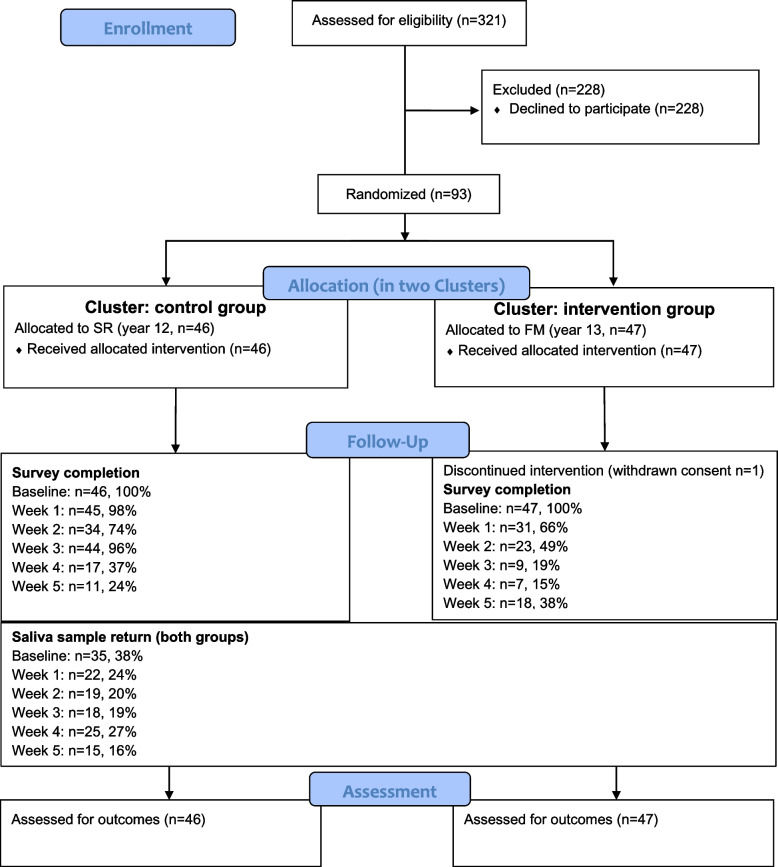
CONSORT diagram. SR, school rules group. FM, face mask group

Ninety-three of 321 eligible pupils (SR = 46, FM = 47) agreed to participate, yielding a recruitment rate of 29% (see Fig. 3). Seventy pupils gave reasons for not taking part in the study. Reasons included unwillingness to wear face masks (*n* = 26, 51%), unwillingness to provide saliva samples (*n* = 11, 16%), no interest in the study or focusing on own study (*n* = 7, 1%), no benefit from taking part or too much effort (*n* = 7, 1%), and not understanding the reasons behind this study (*n* = 3, 1%). Demographic characteristics are summarised in Table 2.

**Table 2:** Demographic characteristics of participants

	SR (control)	FM (intervention)	Total
*N*	46	47	93
Age (mean, SD)	16.1 (0.3)	17.1 (0.4)	16.6 (0.61)
Gender (*n*%)			
Female	20 (43%)	25 (53%)	45 (48%)
Male	23 (50%)	18 (38%)	41 (44%)
Other	< 3	< 3	- (omitted)
Prefer not to say	< 3	< 3	4 (4.3%)
Ethnicity			
White British	18 (39%)	18 (38%)	36 (39%)
Asian	11 (24%)	15 (32%)	26 (28%)
Black	5 (11%)	7 (15%)	12 (13%)
Chinese	< 3	< 3	- (omitted)
Other	8 (17%)	3 (6%)	11 (12%)
Prefer not to say	< 3	3 (6%)	- (omitted)
Vaccine status			
Yes	39 (85%)	35 (74%)	74 (80%)
No	7 (15%)	11 (23%)	18 (19%)
Prefer not to say	< 3	< 3	< 3
Vaccine doses			
1 dose	4 (10%)	4 (11%)	8 (9%)
2 doses	22 (56%)	20 (57%)	42 (45%)
> 2 doses	13 (33%)	10 (29%)	23 (25%)
Household size			
2–3 people	14 (30%)	9 (19%)	23 (25%)
4–6 people	31 (67%)	35 (74%)	66 (71%)
> 6 people	< 3	< 3	4 (4%)

### Adherence: mask-wearing

**Fig. 4:**
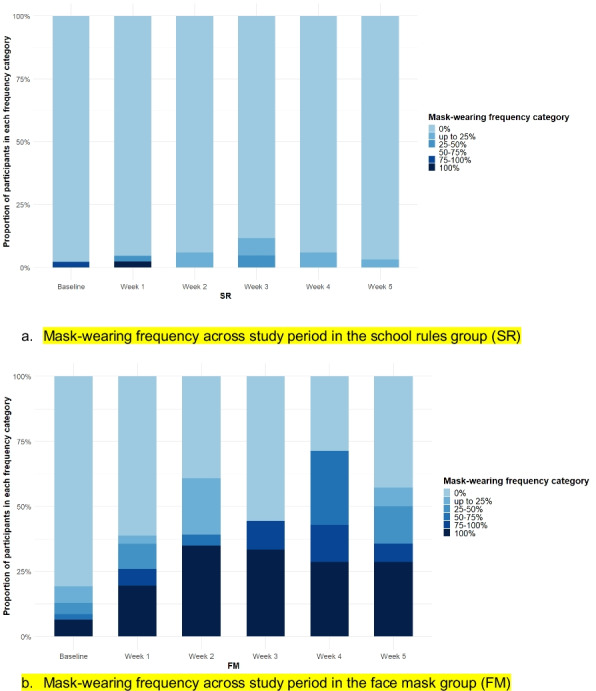
**a, b** Adherence to mask-wearing by weeks and trial groups

In Fig. 4a, b, we show the frequency of mask-wearing behaviour across the 6 weeks of the study, with the SR group shown in Fig. 4a and the FM group shown in Fig. 4b. This shows that, at baseline, none (0%) of the participants in SR and *n* = 8 (17%) participants in FM reported wearing a face mask (across all frequency categories). The number of participants in SR wearing face masks remained relatively unchanged over the trial period (see Fig. 4a). In contrast, the number of participants in FM wearing face masks changed, with fewer than 3 (< 7%) of FM participants reporting wearing a face mask at all times at baseline, reaching an average of 28% (SD = 6.8) over subsequent weeks (see Fig. 4b).

Out of the 33 teachers who participated in the trial,* n* = 10 (30%) reported on the number of pupils wearing face masks in classes, contributing an average of 18 observations over the trial period. This corresponded to an estimated 3% of all classes for FM. These teacher-derived observations of mask-wearing suggested that on over half of all occasions (58%) they reported between 0 and 25% of pupils wearing face masks in class across week 1–5, and 40% of all observations reported “0% of pupils wearing face masks in class”. Another 2% reported “between 25 and 50% of pupils wearing face masks in class”. These findings largely corresponded with the average mask-wearing reported by pupils.

### Adherence: saliva sample collection

**Fig. 5:**
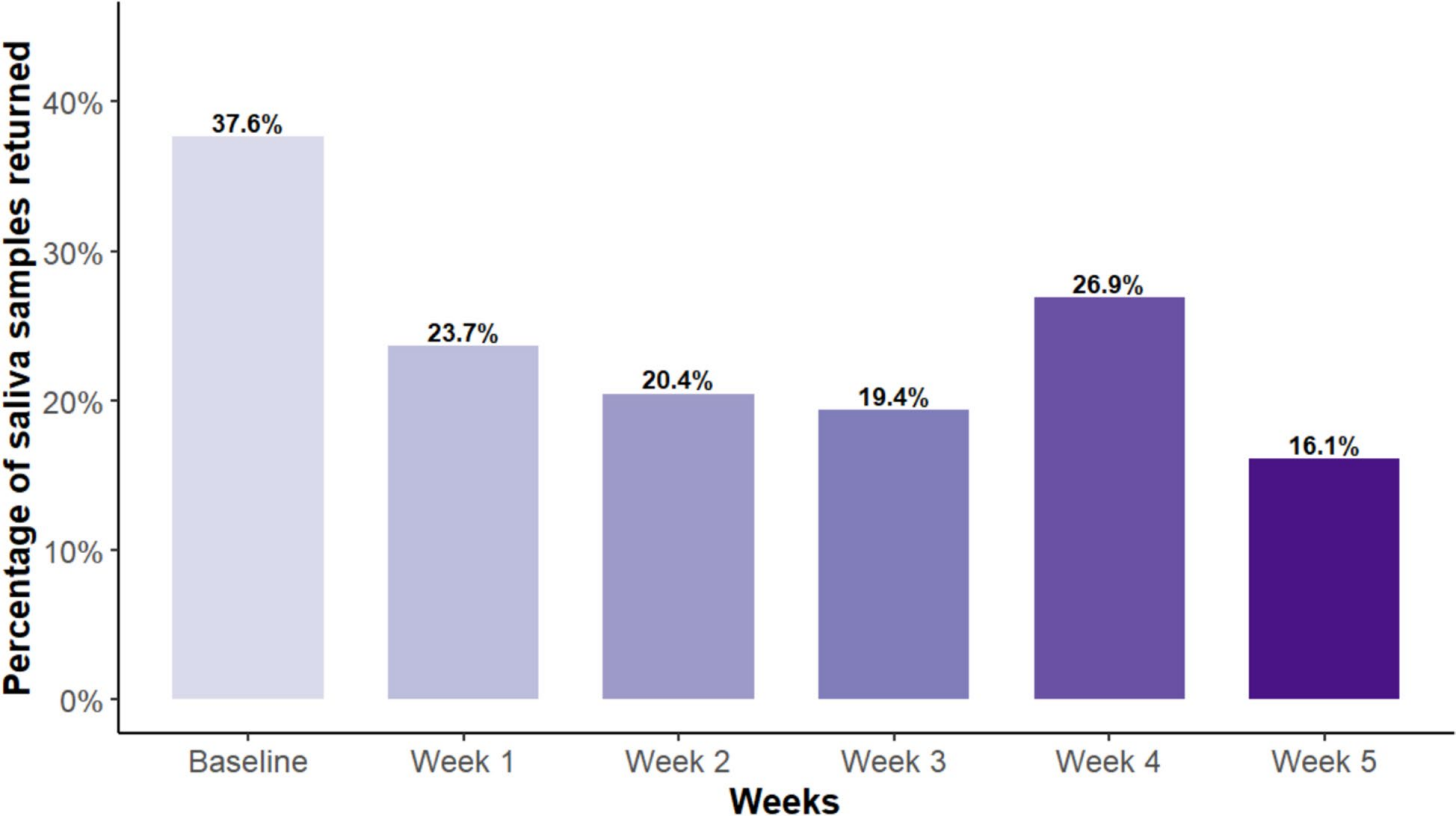
Percentage of saliva samples returned by week

At baseline, *n* = 35/93 participants provided saliva samples. Over subsequent weeks the average number of saliva samples returned was 20 (see Fig. 5).

### Effects of mask-wearing on learning, communication, physical health, and mental health

Over the entire trial period, a total of 62 individual survey responses were received from both groups (51 from FM, 11 from SR), regarding the impacts of mask-wearing on their learning, interactions with people, their overall physical health and mental health. Overall, the majority of pupils reported that wearing face masks did not affect their learning (79%), interaction with friends (73%), interaction with teachers (81%), interaction with other people (87%), physical health (69%), and mental health (77%). The reported median was 3 (not much impact/same as not wearing a face covering), for all of the outcomes, for both trial groups. Summary of the number of participants who reported on these outcomes, and medians (Q25, Q27) are presented in Table 3.

**Table 3:** Summary of participants who reported on the impact of mask-wearing on learning, interactions with people, physical health, and mental health by trial arms

	SR		FM	
Impact of mask-wearing	Responses	Median (Q25, Q75)	Responses	Median (Q25, Q75)
On learning	11	3 (3, 3)	51	3 (3, 3)
On interactions with friends	11	3 (2, 3)	51	3 (3, 3)
On interactions with teachers	11	3 (3, 3)	51	3 (3, 3)
On interactions with others	11	3 (3, 3)	51	3 (3, 3)
On physical health	11	3 (3, 3)	51	3 (2, 3)
On mental health	11	3 (3, 3)	51	3 (3, 3)

*SR* school rules group, *FM* face mask group

Sixteen percent of all responses reported a somewhat or extreme negative impact of mask-wearing on learning. The most common negative impact related to communications in classes (e.g. “*Can’t speak clearly in class*.”, quote from pupil). Ten to 18% of all responses reported somewhat to extreme negative impact of mask-wearing on the interaction with friends, teachers or others. The most common negative impact related to difficulty hearing or communicating with others (e.g. “*Friends can’t understand what I’m saying*.”, quote from pupil). Twenty-nine percent of responses reported somewhat to extreme negative impact on pupil’s physical health. The most common negative impact were skin problems (e.g. rashes, break out, acne), headaches, and worsened migraine related to wearing face masks. For the impact of mask-wearing on mental health, 11% of all responses reported somewhat to extreme positive impacts. The most common positive impacts relate to feeling safe, less self-conscious or anxiety while wearing face masks (e.g. “*People don’t have to look at my face.”,* quote from pupil). Another 11% of counts reported negative impacts of mask-wearing on mental health but no qualitative quotations were given. Nine (15%) pupils reported stigma (discomfort being seen wearing face masks at school) over the trial period, while 46 (74%) reported no stigma.

### Mood

**Fig. 6:**
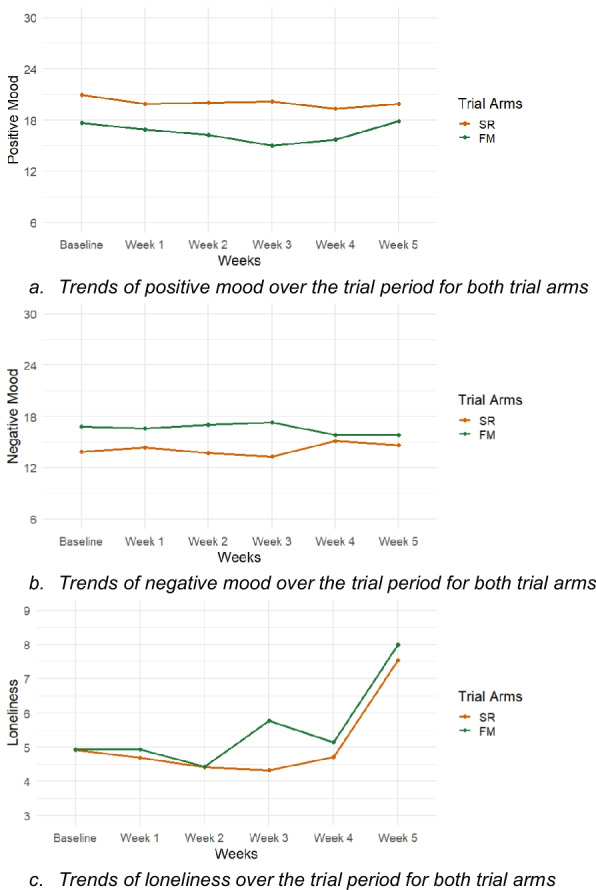
**a**, **b**, **c** Trends of mood and loneliness over the trial period for both trial arms. SR, school rules group. FM, face mask group

Summaries of positive and negative mood, and loneliness scores (means and standard deviations [SDs]) by year groups and weeks are presented Fig. 6 and Supplementary Appendix Table D1. Both measures of mood and loneliness appeared largely stable over the trial period. However, levels of loneliness showed an upward trend nearer the last week of the trial (see Fig. 6c). We did not conduct statistical tests to examine whether there were significant differences in mood and loneliness between groups and over time, thus these results may only provide overall descriptions of mood and loneliness among participants, and information on the potential directions of change.

### End of trial feedback and recommendations

Forty-nine pupils provided feedback on providing saliva samples in the last weekly survey, including 14 pupils from FM and 35 pupils from SR. A summary of the feedback is shown in Supplementary Appendix C. Overall, most pupils found the instructions on how to collect saliva samples easy to understand (76%). Almost half of the pupils (45%) thought that the process was easy. However, the majority of pupils experienced some level of discomfort when collecting the samples (73%).

Pupils were also provided with an opportunity to provide suggestions on how to improve saliva-sample collections in a future trial, using a free-text response. Their suggestions included: using stronger straws (e.g. plastic straws) for collection of saliva samples; allowing participants to collect saliva samples at a more private place (e.g. a cubicle), and providing less saliva.

We further asked pupils’ opinions on whether to include incentives, giving participants a choice for where to collect their saliva samples, and using reusable masks in a future trial. They provided responses to a 5-point scale for each question. One hundred and fifteen pupils answered these questions, including 50 pupils who took part in this feasibility trial and 65 pupils who did not. A summary of these results is shown in Supplementary Appendix C. Overall, the majority of pupils would be more likely to take part in a future trial if they were to receive an incentive (e.g. shopping voucher; 70%), or if they were able to choose where to provide their saliva samples (57%).

## Discussion

We describe here the co-creation activities we undertook to inform the design of a cluster-randomised controlled feasibility trial of face masks in schools, and the results from this trial. Our goal was to develop methods that would enhance recruitment to the trial and engagement with trial procedures, in particular wearing of face masks in the intervention arm. The process of designing the trial involved secondary analysis of existing qualitative data and iterative PPI focus groups. Through these activities we identified barriers and facilitators relating to trial participation and engagement, and theory-based solutions. In the trial we observed a recruitment rate of 29% and, on average, 28% of pupils reported wearing masks at all times in the intervention arm. We also observed considerable variability in the completion of other trial procedures related to providing saliva samples and completing surveys. However, it was also clear that responses to measures of positive and negative mood and loneliness remained highly stable over the course of the trial, suggesting no adverse effects of mask-wearing or trial participation on these outcomes. Engagement in the trial procedures and mask-wearing declined over time, suggesting that initial engagement will wane especially where only a minority of school students participate at the outset.

Regarding recruitment, the rate observed in the present work is lower than reported in face mask trials conducted in non-school settings such as healthcare, households and community contexts (35–86%, [34–36]), and thus was deemed not feasible for a future trial of such design. Indeed, despite efforts to address many of the challenges to mask-wearing identified by young people in our pre-trial PPE work, over 50% of pupils indicated that the main reason for not participating was their unwillingness to wear masks. These findings highlight the need for continued development of innovative approaches to facilitate the recruitment of young people into trials. Recent reviews suggest that social media and use of existing social networks may prove a fruitful avenue for future work [37, 38].

We also observed limited engagement with mask-wearing in those pupils allocated to the intervention arm, and in both groups, with the provision of saliva samples and survey completion. The low levels of adherence and engagement in the trial procedure also indicate that a future trial of such design might not be feasible in school settings. We can, however, gain some insight into the reasons for this and potential solutions from both our PPI work pre-trial and the feedback from trial participants. For example, regarding mask-wearing, there was evidence that young people needed a clear and personally relevant rationale for engaging with this behaviour; that the behaviour of those around them (both staff and pupils) was an important social influence and that practical challenges also exist in the form of effects on appearance and communication and ‘remembering’. Although the majority of pupils thought that mask-wearing did not have much impact on their daily activities such as learning and interactions with others, 10–20% of pupils did identify one or more of these as among the challenges experienced whilst wearing masks. Indeed, our findings also resonate with those reported in previous studies examining barriers to mask-wearing such as difficulty communicating with others, discomfort, and forgetting [39–41]. While we attempted to address many of these barriers (e.g. providing reminders, focus on autonomy, rationale built around social justice), there may be other facilitators that could be considered in future work. For example, pupils indicated that providing incentives such as shopping vouchers for both taking part and engaging with trial procedures could be an effective incentive. Furthermore, pupils who responded to the feedback questions said that giving them choices on where to collect saliva samples (agreed by 57% of respondents), and using reusable face masks (agreed by 30% of respondents) might also attract more pupils to participate in the trial. The use of reusable face masks might, however, not be feasible given the uncertain evidence regarding their effectiveness [12, 42]. However, future studies could seek to provide a clearer rationale for the choice of face masks.

The survey completion rate also fluctuated in this study from 100% for both groups at baseline, reducing to 15–66% in the intervention group and 24–98% in the control group over subsequent weeks. Survey completion rates were higher in weeks where pupils completed the survey together at the school assembly, while lower completion rates were observed when pupils were asked to complete the survey separately in their tutor groups. This suggests that gathering all pupils together at a designated lesson (e.g. an assembly) and allowing them to complete the survey at the same time might help maintain adherence to weekly surveys.

Similarly, adherence with the provision of saliva samples was 38% at baseline and was reduced to an average of 22% over subsequent weeks. As noted above, participants suggested that allowing participants to choose where to collect saliva samples might help to maintain sample provision. This is in keeping with recent review evidence suggesting that the provision of choice can, in some contexts, contribute to significantly less participant drop-out and better adherence [43]. For future trials over a longer period (e.g. 1 year), using other outcomes such as reported infections, self-reported symptoms of infections, number of days of illness due to infection [44, 45] might also be appropriate measures of ARI-related outcomes.

Finally, we also observed limited engagement by teachers with the reporting of mask-wearing by pupils, with only 30% providing these data. However, teachers’ observations largely corroborated pupils’ self-report, suggesting that pupil self-report may be an adequate way to capture this outcome.

This study has a number of strengths and limitations. First, to our knowledge this is the first study to examine the feasibility of conducting a two-armed cluster-randomised controlled trial to examine the effectiveness and impact of face masks in schools. We reported findings that can improve our understanding of mask-wearing in school-aged children, and may improve the design of such trials in the future. Second, we co-created the feasibility trial with the school and representatives of the trial participants including school-aged children, teachers, and parents. These co-creation activities improved the feasibility and effectiveness of our trial approaches. The environment and curriculum at this school may not apply to other schools. Third, this study was conducted roughly 8 months after the removal of most restrictions for controlling the COVID-19 pandemic, during which face masks were mandated during several periods. This might have contributed to a sense of ‘fatigue’ regarding protective measures and mask-wearing in particular [46, 47], and may have contributed to the low participation and adherence rates observed in this trial. It is also worth considering that, as the peak of the COVID-19 pandemic was moving away when this trial was conducted, the motivation to wear face masks in children may have declined over time as the perceived risk might be lower. If a future face mask trial were to be conducted in school settings outside the context of a pandemic, addressing other trial facilitators, such as social influences and norms [48], might encourage participation and engagement.

## Conclusion

Our study showed that a cluster-randomised controlled trial to investigate the effectiveness and impact of face masks in schools is likely to encounter recruitment and adherence challenges as seen in other trial settings. However, our findings highlight ways in which these challenges may be addressed. The broader question that remains, however, is whether future work should prioritise the delivery of a pragmatic trial to assess the effectiveness of face masks in the ‘real world’ of school settings; or a tightly controlled trial in which recruitment and adherence are incentivised and optimised to provide a more robust estimate of the efficacy of masks in school-aged children. Clamour for either or both types of trial is likely to continue as long as the effects of prolonged mask-wearing in this population remain poorly understood (e.g. social and educational harms); school-aged children remain vectors for the spread of infection into the community and there remain few other interventions available to schools to reduce the spread of infection.

## Supplementary Information

Supplementary Material 1. Supplementary Appendix A: Table A1. Themes and subthemes from secondary analysis. Supplementary Appendix B: Registration survey for the Patient and Public Involvement (PPI) focus groups. Table B1: Summary of barriers and facilitators identified in the secondary analysis and PPI focus group and proposed solutions and associated theoretical component. Table B2: Summary of PPI focus group findings. Supplementary Appendix C: Example of guidance on the appropriate way to wear a face mask. Supplementary Appendix D: Table D1. Mood by year groups and weeks.

Supplementary Material 2.

## Data Availability

The datasets used and/or analysed during the current study are available from the corresponding author on reasonable request.

## Abbreviations

ARI: Acute Respiratory Infections
FM: Face Mask Group
PPI: Patient and Public Involvement
RCT: Randomised Controlled Trial
SR: School Rules Group
TeC-19: Study Teenagers’ Experiences of the COVID-19 Study
WHO: World Health Organization
