# Supplementary material for: Understanding and addressing the obstacles to a trial of face masks in schools: a mixed-methods feasibility study
Source: Pilot Feasibility Stud. 2026 May 22;12:101. doi: 10.1186/s40814-026-01825-7 (PMC13383452; doi:10.1186/s40814-026-01825-7)
Supplement: Supplementary file 1 — Supplementary Material 1. Supplementary Appendix A: Table A1. Themes and subthemes from secondary analysis. Supplementary Appendix B: Registration survey for the Patient and Public Involvement (PPI) focus groups. Table B1: Summary of barriers and facilitators identified in the secondary analysis and PPI focus group and proposed solutions and associated theoretical component. Table B2: Summary of PPI focus group findings. Supplementary Appendix C: Example of guidance on the appropriate way to wear a face mask. Supplementary Appendix D: Table D1. Mood by year groups and weeks. [file 40814_2026_1825_MOESM1_ESM.docx]

**Understanding and addressing the obstacles to a trial of face masks in schools: a mixed-method feasibility study**

**Supplementary Appendix**

Contents

[Supplementary Appendix A 2](#_Toc225266873)

[Themes and subthemes from secondary analysis 2](#_Toc225266874)

[Supplementary Appendix B 10](#_Toc225266875)

[Registration survey for the Patient and Public Involvement (PPI) focus groups 10](#_Toc225266876)

[Topic guides for the Patient and Public Involvement (PPI) focus groups 12](#_Toc225266877)

[A. Topic guide for young people 12](#_Toc225266878)

[B. Topic guide for teachers/parents 16](#_Toc225266879)

[Summary of PPI focus group findings 20](#_Toc225266880)

[Table B1. Summary of barriers and facilitators identified in the secondary analysis and PPI focus group and proposed solutions and associated theoretical component 20](#_Toc225266881)

[Table B2. Summary of PPI focus group findings 41](#_Toc225266882)

[Supplementary Appendix C 66](#_Toc225266883)

[Example of guidance on the appropriate way to wear a face mask 66](#_Toc225266884)

[Example of guidance on how to collect a saliva sample 67](#_Toc225266885)

[Example of a weekly survey 68](#_Toc225266886)

[Trial Procedure and Adjustments 77](#_Toc225266887)

[Summary of results for end of trial feedback and recommendations for future work 79](#_Toc225266888)

[Supplementary Appendix D 80](#_Toc225266889)

[Table D1. Mood by year groups and weeks 80](#_Toc225266890)

# Supplementary Appendix A

## Themes and subthemes from secondary analysis

Table A1. Themes and subthemes from secondary analysis

| **Theme** | **Subtheme** | **Quote** | **Description** |
| --- | --- | --- | --- |
| Barriers to wearing face coverings and masks | Beliefs about effectiveness | I don’t think face masks… like they will work if people have it, but if people don’t have it, it’s kind of stupid and almost like increases your risk, ‘cause the virus is gonna get on your mask, you’re gonna touch the mask when you take it off.  I do agree that like, obviously it’s good to make people feel safer, but also think it gives people like a bit of a like false sense of security  it’s like it doesn’t really work, like I think people think it works a lot more than it does, but I guess if it makes them feel better | Some people thought that face masks gave a false sense of security and could even increase the risk due to touching the mask. |
|  | Beliefs about susceptibility to Covid-19 | To be honest – like, cos if I’m gonna be honest, it doesn’t bother me because I’m a person who’s not at risk. I have no illnesses, I’m young.  I feel like, ‘cause we’re not really the at-risk group, it kinda feels at the same time that all this is unnecessary for someone that’s probably not gonna… that’s probably gonna have the like the mildest symptoms, sort of like what’s the point? | Those that believed that they were at low risk of COVID seemed more likely to say that they may not wear a mask or be less likely to be bothered by others not wearing masks – especially if only really mixing with each other. |
|  | Discomfort and appearance | A lot of us have found it like quite annoying, like especially on the bus. So today it was like twenty-seven degrees and we were on with our masks and it was like everyone was just so sweaty and the ?air con? wasn’t working so we were on there for like just under an hour and it was horrible.  And it definitely did get very annoying after like a… like a couple of weeks of wearing it, because like you would all feel stuffy and stuff like that and… I don’t know, it definitely got very annoying, and I didn’t like going out for long periods of time when I was wearing them | Several people mentioned issues related to discomfort or appearance, such as being uncomfortable when hot, looking ‘uncool’, worries about making acne worse, not being able to hear others well and glasses steaming up. |
|  | Social influences | Don’t really social distance at all, to be honest. We just get in the car and go with, like, whoever we want. I mean, I’m just being honest. Like no-one does it. No-one wears masks.  You look around and no ones wearing one | Social influences were mentioned as potential barriers. |
|  | Confusing or inconsistent rules | I have no idea, but it does seem a bit weird that all the passengers have to do it but they [bus drivers] don’t.  Like, it’s a bit like just confusing and complicated, cos for some things you do have to wear a mask, for some things you don’t have to wear a mask. It’s not very clear. | People also talked about confusion and inconsistency in terms of rules at various points over the pandemic. |
|  | Forgetting | And sometimes I’ll forget to do it as well, so. ‘Cause like I don’t see anyone with it so I’m not reminded and then, yeah.  We’re all following what’s been put in place, but there’s little things, like we wear our masks in the corridor, but there’s always that one person that gets stopped because they’ve like forgotten to put their mask on | Some people reported forgetting their masks if not reminded. |
|  | Masks aren’t enforced | I think it’s a bit flawed because some places are, like, quite strict on it and some places are not.  Cause at our school they say that they recommend you wear a mask so it’s not enforced so a lot of people don’t.  It’d be very hard to enforce as well. It’s like outside (Overtalk), they’re not gonna have the police everywhere are they, so a lot of people just wouldn’t bother, even if it was guided. | In terms of enforcement of masks, with many saying that when they don’t have to wear them or when masks are not enforced they are less likely to wear them. |
| Facilitators to wearing face coverings and masks | Adhering to rules | Well I don’t use one at the moment, but if it’s… if they tell me to do it, like I’ll do it.  We will bring masks and we do wear them when we were, like, in the queues and stuff, and cos obviously you have to. | Some people reported wearing a mask because they were told to, or had to. |
|  | Social pressures and influences | Yeah, it’s bad because like I didn’t want to wear a mask when you didn’t have to just because I felt a bit silly, but now that, you know, a majority of people are wearing it, it’s definitely… You know, you feel a lot less… like you don’t feel like you’re sticking out like a sore thumb (laugh). So yeah.  I also had one, ‘cause my parents would make us like wear them when we went into Marks and Spencer’s or whatever.  There’s definitely peer pressure ‘cause you definitely look like an odd one out if you’re not wearing it  But I think it’s good because it’s, people seem less uneasy  worn a face mask since the start of lockdown was when I was helping my great grandma move house, like moving all her furniture, ‘cause she lived in like a community home, so we had to definitely wear masks for that. | Social influences/pressures were also important as facilitators. Social influences ranged from parents telling them to wear a mask, to friends – not wanting to look like the odd one out, to the general public – i.e. its easier when everyone is wearing one, acts as a reminder. Also, some mentioned wearing masks to protect vulnerable others. |
|  | Beliefs about effectiveness and safety | Obviously masks aren’t the most comfortable, but they keep us safe.  But then it’s like I have to wear it, otherwise there’s a chance I might get the virus.  Well, I think it’s good because, obviously, it’s been proven to like reduce spreading | Several people cited that they wore a mask because they believed them to be effective in keeping them safe and protecting others. |
|  | Comfort/ease | But at the same time, you could argue it’s quite an easy thing to do.  I don’t really mind because my mask is, like, quite comfy and it doesn’t really get that hot  And also it’s alright cos no-one can really tell who you are when you go to the shops, so you don’t really have to put an effort in when you go to the supermarket or anything. | Some people said it’s easy, and some found masks comfortable. |
|  | It’s the new normal | It’s sort of, I don’t know, it’s pretty much in your head now that you’ve gotta wear a mask everywhere.  which I think if we were to be wearing them for the next, like, four or five years, which I don’t necessarily – I think it will eventually get to that point where it’s a habit, an expectation, and if you don’t have it it’s just a weird thing. I don’t know. | Several people also noted that it’s the new normal, so it’s just ‘in your head now’. |
| Indifference/they’re not great but not bad |  | Well, it’s a good thing cos we get to, like, go out again, but… and to be honest I don’t think – the masks don’t really bother me. They’re not like that annoying. It’s just like a way to prevent it, isn’t it?  I had to wear one when I was at the barber’s, I think it was last week or the week before, whenever they opened, and they’re not that bad. | There are a group in the middle, who report either not being that bothered/indifferent to masks, or that they don’t particularly like them but are still willing to wear them. |
| Face coverings in schools | Variability in rules and usage in schools | We only have to wear them in the corridors so it’s not like… a lot of the routes that you sort of made are outside so a lot of the time you don’t actually, unless you’re going upstairs, you don’t really have to wear them very, like for a long period of time  So we have to wear a mask at all times in the building, even in the classroom, which is I know is different to a lot of schools.  Well there’s a few people in my class that don’t wear their masks, or like when they… we… when we walk into the class we have to, our temperature has to get checked, and sometimes like they dodge that somehow or like they don’t even put hand gel on.  I mean I’ve not really seen anybody who’s just… I’ve not really seen anyone refuse to wear one. They have them available, if you’ve not got one with you or you’ve forgotten one, they do have them available to like get for you. But I don’t really think I’ve seen anybody like refuse to wear one as of yet, so. | Most people reported that they had to wear them in corridors but not classrooms, whereas for a minority they had also worn them in classrooms.  Variability between schools in enforcement of masks and also variability in how prevalent mask wearing was with some reporting nearly all pupils wearing them, and others reporting a culture of students finding ways around the rules and it not being enforced by teachers because ‘they can’t make you’. |
|  | Mask wearing at school depends on context or risk beliefs | I’ve had my mask on in like some of my lessons, which I’m not like… which I’m like directly next to people if I’m... (Stutters) I cannot speak. If I’m in my large classes, like for psychology. But for my smaller classes, like my biology class, it’s… I’m like… there’s a chair, there’s a seat in between me and the next person, so I’m not really wearing my mask in there. | Some wore masks in classes where they felt more at risk because of not being able to distance, or only in places where it was necessary. |
|  | Mask wearing increases feelings of safety at school | I do feel safe whilst I’m at school, but especially the day before I was going, I heard on the news that there was nearly three thousand new cases in the day prior, so that made me a little bit uneasy, but… which like made me have my mask on, like I’ve had my mask on in like some of my lessons | This one was only mentioned by one participant, but they felt that when cases were rising, wearing a mask at school made them feel a bit safer. |
| Face mask usage | Variation in attitudes | I think that we… in our group we’ve got quite a bit of a range of, like, attitudes towards trying to avoid the virus. Like there’s people that don’t care at all, which I probably fall under, to be honest, and there’s people that actually actively try, go out and wear masks and whatever. | Divergent attitudes towards face masks, with some saying that they were important, and that they liked them, and others saying that they were really disliked. Also evidence of conflicting views with some saying they’re annoying but also seeing the benefit. |
|  | Impact of time/change in use and attitudes over time | People aren’t scared enough anymore and just there’s too many people that are no longer worried about it.  I don’t really understand why, like – obviously shops opened on the 15th of June when the virus was way worse, why we didn’t have to wear a mask then, but we do now, and surely the risk has gone down? It just seems a bit silly that they didn’t introduce these measures when it was worse. Like, I don’t really understand. | Time also seemed to have an impact with many reporting that the mandatory face covering rule came in too late and would have been more effective when everyone was more scared. Similarly, views were expressed that the longer that the pandemic went on and people were not affected, masks were felt to be less relevant. |
|  | Variations in reported usage | I’d say, like, a small proportion of people actually, like, I’d say they would be lying if they don’t actually stick to the rules, like, properly. Cos well, you look around and no-one is, like, really wearing masks and no-one’s really keeping that distance like they should.  But then as soon as, like, I started coming out of the house I would always wear one. I had, like, a washable one. I dunno, it’s cool, so I always used to wear it. | Usage also varied between participants, with some saying that they always wore a mask and did so even before it was mandatory, whereas others said they only did when they had to. |
|  | Impact of the context | I think my point is more about in places like a restaurant or a pub it becomes a bit weird. I don’t know, just in my experience of going to the pub, trying to think about sitting there with your friends with a mask on.  We will bring masks and we do wear them when we were, like, in the queues and stuff, and cos obviously you have to. But then at, like, Thorpe Park there’s barely any social distancing going on with, like, everyone there. But like in the cars, yeah, we don’t really wear masks | Context was important, for instance wearing masks in shops and on the train or school bus was generally accepted, whereas many felt that it was unnecessary or pointless in places like pubs and restaurants. |
|  | Impact on other measures | I just think keeping social distancing’s better than actually wearing a mask  I suppose it’s better than having like no social distancing, because it means there’s less of a chance of it spreading around the school, I guess.  I have a theory that they’re only bringing it in now because they might abolish social distancing, or reduce it even more in supermarkets.  like they’ll come really close to you because they’re wearing a mask | Discussions about face masks compared to other measures, such as whether wearing a mask was better than social distancing or not and whether they would be necessary once they had been vaccinated. |
| Identity | Importance of friends and socialising | I feel, like when you’re thirty it’s probably nowhere near as critical for you to be with your friends as when you’re eighteen.  The fact we’ve missed like our whole eighteen, well for most of us anyway, I think that’s like a big age in our life where you can like socialise, meet new people, stuff like that. Not stay in your room, play Fortnite or whatever, drink. It’s a bit depressing. | A lot of discussion about the importance of socialising, especially for those who had turned 18 during the pandemic who felt that they were missing out on an important year. |
|  | Young people are blamed | I hate that the young people are being scapegoated for the rise in cases, when they literally incentivised us to go out and eat.  I don’t feel targeted when I’m out, I feel much more targeted like online, just from like the media, because it’s like I feel like we’ve been put in a trap, ‘cause it’s like they… they’re urging us to go out, but I know it’s already been said, but it’s like they’re like blaming us all of the time for, you know, going out too much | A general feeling that young people were blamed or scapegoated. |
|  | Young people aren’t a priority | I just don’t think we’re really the most important at the moment, because there’s people that are working that are important to drive the economy, and I think it’s what the economy needs. We’re… we’re just… we’re at school, and we don’t have exams, like we’re not really the priority. | Young people weren’t a priority throughout the pandemic. The focus was either on older adults or vulnerable people. Working adults, or school aged children were not considered. |

# Supplementary Appendix B

## Registration survey for the Patient and Public Involvement (PPI) focus groups

| Questions | Answer options (if apply) |
| --- | --- |
| 1. Do you live in the UK | Yes |
|  | No |
| 1. Are you… | Aged 16-18 years in full-time education (the equivalent of years 12 or 13 in England) |
|  | A teacher with responsibility for young people 16-18 years old in full-time education |
|  | The parent of a 16-18-year-old in full-time education |
| 1. Email address |  |
| 1. Name |  |
| 1. (for young people only) Are you in… | Year 12 |
|  | Year 13 |
| 1. (for young people only) What is the name of your school |  |
| 1. (for young people only) Which city or town are you from? |  |
| 1. (for young people and teachers only) Do you currently wear any type of face-covering (e.g., a face mask) while in the classrooms? | Yes, always |
|  | Yes, most of the time |
|  | Yes, occasionally |
|  | No |
| 1. Would you describe yourself as... | Anti-face masks |
|  | Pro-face masks |
|  | Not bothered either way |
| 1. When you are not wearing a face mask, it is because... (Please tick all that apply) | I don't want to wear them |
|  | I find them uncomfortable |
|  | I find them inconvenient |
|  | I don’t think they work |
|  | They attract unwanted attention/comments |
|  | I find it difficult or impossible to communicate while wearing a mask |
|  | I find it difficult or impossible to do my job while wearing a mask |
|  | I don’t like how they look |
|  | I can never get hold of them |
|  | I can’t afford them |
|  | I didn’t have a mask with me |
|  | I have a disability that prevents me from wearing a mask |
|  | I am medically exempt from wearing a mask |
|  | Other (please specify) |
|  | None of the above |
| 1. How would you describe your gender | Female |
|  | Male |
|  | Non-binary |
|  | Gender-fluid |
|  | Prefer not to say |

## Topic guides for the Patient and Public Involvement (PPI) focus groups

### A. Topic guide for young people

Pre-amble:

- Thank participants for reaching out and expressing interest in research
- Explain purpose i.e., these are very informal discussions which are a standard step before starting formal research. It will inform what that research will look like, a little bit like market research, to make sure we’re asking questions in right way and looking at the right issues – that’s why we haven’t asked you to complete consent forms or go through other more formal processes
- The point of these discussions are to help us prepare for a future trial that will look at how face masks are effective in protecting in schools from infection – most familiar at the moment with Covid, but we’re also interested in other illnesses, like colds and flu. One of the issues – a lot of good evidence but comes from what we call observational studies, but people haven’t experimentally been put into groups at random – so possibility that the effect is nothing to do with facemasks – so trials are the best method in medical research. No current trials in schools. So we want to talk to you about your opinions on face masks whether pro or con, and also your thoughts on a trial
- Confidentiality statement/withdrawal
- Going to be recording – just for our purposes, won’t be shared, help us with note taking
- Encourage camera on – we’ve found it takes a lot longer if people mainly use the chat function to take part, so if you are interested in sticking to time then I really would encourage you to have your camera on and participate over the microphone
- House rules – please respect each other, try not to interrupt or talk over others, even if your views differ or you don’t agree with what’s being said
- Vouchers – please email the faceit email address to confirm your full name and that you’ve taken part today, then you should receive voucher within a few days – if not do email again. If not interested in taking part in future research, also email us to let us know
- Opportunity to ask questions

Opening questions:

1. What are the first word/words that comes to mind when you think of face masks?
2. What are the first words/thoughts that comes to mind when you think about wearing masks in schools?
3. Do you currently or have you had to wear face coverings at school over the past 12 months? How would you describe your experience?
   1. *[summarise some of the negatives they come up with]*
   2. Are there are any other reasons which might make you:
      i. refuse to wear them
      ii. be reluctant to wear them
   3. *[summarise the positives]*
   4. Are any other reasons which might make you:
      i. definitely agree to wear them
      ii. agree reluctantly

*Probe only if needed: are they generally enforced at school; do most people follow the rules; are rules clear*

Perceptions of face masks over time:

1. How important do you think face coverings are and why?
   1. Can you tell me about any other reasons, situations or factors which influence how important you think masks are?

*Prompts:*

- - 1. *being vaccinated and whether vaccines are effective*
    2. *understanding of covid*
    3. *emergence of new variants*
    4. *having had covid*
    5. *your own risk changes*
    6. *risk of friends or family changes*
    7. *being in spaces indoor and outdoor you can’t be distant*
    8. *being in indoor places not well ventilated*
    9. *beliefs about effectiveness*
    10. *beliefs about susceptibility/risk and seriousness of illness*
    11. *your ability to minimise risk of infection*

1. Next I am interested in how your opinion has changed about face masks over the course of the pandemic?
   1. What did you think when they were first introduced in schools?
   2. Since vaccines were introduced
   3. When they were mandatory vs optional
   4. When they have been reintroduced most recently

Perceptions of the trial procedures:

These next questions are about a trial that we are planning in the future. We want to do a trial to have a look at the effectiveness of face coverings in schools in preventing COVID-19, as well as other acute respiratory illnesses like colds and flu. At the moment there is no trial evidence in schools, and the evidence outside of schools is a bit inconclusive. One of the biggest issues with the trials in general is getting participants to engage in the trial, which in the case of these studies means usually to wear the masks as requested.

So, our current plan is to first do a small trial in just two schools with pupils in years 12 and 13. This will involve pupils in one to being asked to wear a face covering whilst at school for 6 weeks, and pupils at the other school will follow whatever the government guidance is at that time. Once a week pupils will be asked to provide a saliva sample so that we can measure the number of colds, flu and COVID-19 cases. What we want to do in this small trial is to see if we can develop an approach which increases the chances of people participating by wearing face coverings for 6 weeks and providing saliva samples once a week.

So we’re interested in your thoughts on this initial study. We want to get your ideas on how you might run a study like this. So please don’t worry about offering alternative suggestions or being critical of our current plans. We consider you to be the experts on what would work best in schools.

1. So, first of all, what, if anything, would you want to know about the study before you took part?
2. Thinking about a trial like I described happening in your school:

what would be your first thoughts?

what would make you keen to participate?

what would make you less keen?

are there other factors that you think might be important in your decision to take part

*Probe only if needed: your parents, your friends, the rates of infections at the time, benefits to you, benefits to others; forgetting; supply; discomfort*

Do you think you would take part?

Now let’s talk about wearing masks for 6 weeks – that will be about half a term:

what would be your first thoughts?

what would make you keen to wear a mask throughout this time?

what would make you less keen to wear a mask throughout this time?

are there other factors that you think might be important

*Probe only if needed: things that would make it easier/harder - your parents; your friends; the rates of infections at the time; benefits to you; benefits to others; Particular lessons, classrooms, tasks; type of masks; availability of masks; benefits to others; forgetting; discomfort; having times you don’t have to wear a mask?*

1. What about anything that might help to make it easier to wear a face mask at school?
2. Can you think of any incentives might encourage wearing face coverings for the full six weeks?

*Probe after part a:*

- - 1. *Working as a team*
    2. *Competition between classes*
    3. *Other ideas*

1. How would you measure face mask use in a study like this?
2. As part of the trial, we are hoping to use saliva samples to measure a range of infections like flu, colds and COVID-19. This involves spitting or dribbling though a straw into a sample tube until it is at least half full. At the moment we are planning on asking young people to do this once a week. But we are interested in how you would get people to provide saliva samples if you were designing the study.

Thinking about these saliva samples, what, if anything, would you want to know about providing saliva samples before you took part?

1. What might make you less willing to do this part of the study?
2. Where would you prefer to do it?
3. What would help as a reminder?
4. How many times per week would be acceptable?
5. *[ask about the terms ‘spitting or dribbling’ – what is their reaction to these words, are there better words we could use]*
6. How do you think the study should be advertised for young people?

*Probe only if needed: i.e. any particular aspect to focus on – benefits/returning to normal/helping others*

- Vouchers – please email the Face It email address to confirm your full name and that you’ve taken part today, then you should receive voucher within a few days – if not do email again. If not interested in taking part in future research, also email us to let us know

### B. Topic guide for teachers/parents

Pre-amble:

- Thank participants for reaching out and expressing interest in research
- Introductions
- Explain purpose i.e., just to reassure you these are very informal discussions which are a standard step before starting formal research. It will inform what that research will look like, a little bit like market research, to make sure we’re asking questions in right way and looking at the right issues – that’s why we haven’t asked you to complete consent forms or go through other more formal processes
- The point of these discussions are to help us prepare for a future trial that will look at how face masks are effective in protecting in schools from infection – most familiar at the moment with Covid, but we’re also interested in other illnesses, like colds and flu. One of the issues – a lot of good evidence but comes from what we call observational studies, but people haven’t experimentally been put into groups at random – so possibility that the effect is nothing to do with facemasks – so trials are the best method in medical research. No current trials in schools. So we want to talk to you about your opinions on face masks whether pro or con, and also your thoughts on a trial
- Confidentiality statement/withdrawal
- Going to be recording – just for our purposes, won’t be shared, help us with note taking
- Encourage camera on – tends to work a bit better and run quite a bit smoother if we’re chatting over the microphone rather than the chat feature we’ve found it takes a lot longer if people mainly use the chat function to take part, so if you are interested in sticking to time then I really would encourage you to have your camera on and participate over the microphone
- House rules – please respect each other, try not to interrupt or talk over others, even if your views differ or you don’t agree with what’s being said
- Vouchers – sometimes email addresses don’t marry up to peoples teams’ names, so please email the face it email address to confirm your full name and that you’ve taken part today, then you should receive voucher within a few days – if not do email again. If not interested in taking part in future research, also email us to let us know
- Opportunity to ask questions

Perceptions of wearing face coverings over time:

1. What are the first words/thoughts that comes to mind when you think about students wearing masks in schools?
2. [FOR TEACHERS ONLY] What’s your current experience with face masks in school settings?
   1. Where do they have to be worn?
   2. What has the reaction of students been like?
   3. Are the rules the same for teachers and students?
   4. How has your experience face masks in schools changed over the course of the pandemic?
3. Do you think your pupils/teenagers were or are aware of your views on face coverings? If so how?
   1. If so, do you think it mattered to them/affected their willingness to wear masks?
4. a) Do you think current attitudes to face masks in school have changed amongst pupils since they were first made mandatory?

*Probe if needed: have they been more or less willing to wear them?*

b) Why do you think there has been a change:

*Probe if needed:*

1. *pupils being vaccinated*
2. *family members being vaccinated*
3. *more people having had the infection already*
4. *new variants emerging*
5. *changing views of peers or parents*
6. Thinking about the last time/current guidance to wear face coverings in your school:

[*want to differentiate between when masks were enforced/mandatory and when they are just encouraged – link back to Q2 and discuss separately if needed*]

1. how compliant where your year 12 and 13 pupils in general?
2. what worked best and why?
3. what seemed not to work well and why?
4. were there any pupils who repeatedly did not engage and if so why do you think this happened?
5. Thinking about any other campaigns you have run in your school involving year 12 or 13 pupils (e.g. keeping grounds tidy, anti-bullying campaigns etc.)
   1. can you tell me a little about what the campaigns were?
   2. what helped get year 12 and 13 engaged?
   3. what did not work at all?

These next questions are about a trial that we are planning in the future. We want to do a trial to have a look at the effectiveness of face coverings in schools in preventing acute respiratory illnesses like colds and flu and of course COVID-19, At the moment the trial evidence is limited by one key factor, that most trials have found it difficult to get people to wear masks. This obviously makes it difficult to determine how effective they are. This is the big problem we are hoping to address in our future work.

So, our current plan is to first do a small trial in just two schools with pupils in years 12 and 13. This will involve pupils in one to being asked to wear a face covering whilst at school for 6 weeks, and pupils at the other school will follow whatever the government guidance is at that time. Once a week pupils will be asked to provide a saliva sample so that we can measure the number of colds, flu and COVID-19 cases. What we want to do in this small trial is to see if we can develop an approach which increases the chances of people participating by wearing face coverings for 6 weeks and providing saliva samples once a week.

What we’re really interested in learning from you today is what you think would work best? How would you design a study like this?

So, thinking about a trial like that happening in your school / your teenagers’ school:

what would be your first thoughts?

What might encourage schools to participate (for teachers only)

What do you think might encourage students to take part?

How do you think your views might affect your children’s (for parents only)

Now let’s talk about wearing masks for 6 weeks – that will be about half a term:

what would be your thoughts about how willing your students/children would be to do this?

can you think of things that might encourage them?

Can you think of things that might discourage them?

What about particular strategies to encourage students to stay engaged for the full 6 weeks?

are there other factors that you think might be important?

1. What about anything that might help to make it easier for students to wear a face mask at school?
2. Can you think of any incentives might encourage students to wear face coverings for the full six weeks?

*Probe after part a:*

- - 1. *Working as a team*
    2. *Competition between classes*
    3. *Face mask buddy who helps the other person stick with the masks*
    4. *Influence of teachers, parents or peers*
    5. *Other ideas*

*Probe only if needed: would it be effective, would it be easy/problematic to implement?*

1. How should a trial like this be introduced to staff?
2. What will staff need/want to know
3. Best method of communication
4. How should a trial be introduced to students?
5. What will students want/need to know
6. Best method of communication
7. What might make it difficult for a school to take part in the trial?
8. Any specific lessons/environments where it would be hard to encourage mask use?
9. Any times of the year where engagement in the trial/with face coverings would be harder?
10. [FOR TEACHERS ONLY] How would you measure face mask use in a study like this? For example we want to be able capture how many students were them, where and how often.
11. [ASK IF THERE’S STILL TIME] As part of the trial, we are hoping to use saliva samples to measure a range of infections like flu, colds and COVID-19. This involves spitting or dribbling though a straw into a sample tube until it is at least half full. We know lots of schools struggled with the logistics of LFDs. We hope providing saliva samples would be a lot easier as they would only be asked to do it once a week, and they can be done at home and brought in to school for sending to the lab or could be done at school. Given your experiences of LFD, what would be the ideal procedure to make it as simple as possible for schools and pupils to provide these samples and send them to us?
    1. What do you might encourage students to provide saliva samples at school once a week?
    2. What issues might students have?
    3. Would students providing saliva samples at school once a week be feasible for staff to manage?
    4. What support should be in place/what would you need to help facilitate this?

## Summary of PPI focus group findings

### Table B1. Summary of barriers and facilitators identified in the secondary analysis and PPI focus group and proposed solutions and associated theoretical component

| 1 | **Behavioural Analysis of findings from PPI focus groups and secondary analysis of existing focus group data** | **Category (Methodology/Intervention components)** | **Link to theory (or design issue where indicated)** | **Proposed solution** |
| --- | --- | --- | --- | --- |
| 2 | **Target behaviour: Recruitment into the RCT** |  |  |  |
| 3 | **Barrier: Fear of covid has reduced**  Secondary analysis:  Face mask usage –> Impact of time/change in use and attitudes over time  *“People aren’t scared enough anymore and just there’s too many people that are no longer worried about it”*  PPI focus groups:  Barriers to face masks -> Perception of importance of masks –> Students might not see any difference to them when wearing/not wearing masks  *“It’s because situation is been really, being told things are changing, and then they’re not really seeing any change, …, they’re not seeing any difference wearing a mask and not wearing a mask, so they’re probably a bit like ‘well I don’t know what the point is anymore’*” (Teacher) | Intervention components | Protection motivation theory would suggest current perceptions of risk from covid have gone down (construct = threat appraisal), so this would no longer be persuasive. Therefore, we considered drawing on future risk (threat appraisal) instead. | The trial would not focus on COVID-19, but future risk (and other benefits of participation, see below). |
| 4 | **Barrier: Feeling of being forced to take part in the trial**  PPI focus groups:  Students taking part in the trail -> Barriers -> Don’t like feeling forced  *“You don't feel you're being forced to do it”* (Young people)  **Facilitator: Understanding the importance of the trial**  PPI focus groups  School takin part/Engagement of staff –> Facilitators –> Explaining importance of the rationale of the trial  “*I think, like with the pupils making it really obvious the purpose of it and the significant of it and why it’s being done and why it’s important”* (Teacher)  **Facilitator: Communicate the research with students and parents clearly and transparently, making sure they understand the research and had the opportunity to ask questions before taking part.**  PPI focus groups  Student taking part in the trial -> Facilitators –> Having researchers visit the schools to talk about the study  “*I think that would be an in person thing…I think it would be at class level talking to them and talking to then like an adult. Being honest with them about what it’s about.…they want the proper rationale*.” (Parent)  “*You should, someone should like be, should have an insider like to talk to students*” (Young people)  PPI focus groups  School takin part/Engagement of staff –> Facilitators –> Transparency of what’s involved  “Just kind of like being transparent on details and rationale, I think would be really important” (Teacher) | Intervention components | Self-determination theory would suggest to support choice in order to support autonomous motivation to participate in the trial. | The research team would communicate the research with students and parents clearly and transparently, making sure that they understood the rationale for taking part in the research (i.e. the benefits of participation) so that they can make up their own mind about whether to take part, and had the opportunity to ask questions before taking part (Letters and Live Q&A). A few key strategies included:  • Addressing issues raised in focus groups (e.g. discomfort, appearance)  • Q&A sessions for reassurance and further explanation (face-to-face for pupils; can be virtual for parents)  • Allowing parents and pupils to respond to invitation letters in parallel to allow informed family discussions  • Using different language tailored to different audiences (school/teacher/pupils) when explaining drivers for participation:  o School or pupils were not considered in the pandemic  o Thinking about being prepared for any future pandemic situations  o No previous research/trials conducted in school setting |
| 5 | **Barrier: Parental consent viewed as important for participation.**  PPI focus groups  Students taking part in the trial -> Barriers –> Parental restrictions/consent  *“I think it would need parental consent*” (Young people)  *“I’d probably take part but parental awareness is still more needed*” (Young people)  *“I think you’ll have a few parents who will say no anyway, ‘cause there’re always some parents who are very wary of any data being collected on their children, and when they are asked the question, err on the side of caution”* (Parent)  Expert discussion meeting  “If school meets agreed threshold of consent then go ahead” (Expert) | Methodology | Of relevance to recruitment strategy | Ideal population would be pupils older than 16 (so able to consent themselves)  Introductory information (letters, meetings) would be provided prior to inviting pupils to consent to the project. |
| 6 | **Barrier: Inappropriate timing of the trial (during summer when wearing masks are more difficult or during exam season when pupils don’t have time) or inappropriate groups of participants who might not be willing to take part.**  PPI focus groups  Other issues considered –> Summer would be more difficult in terms of timing -> more complaints about being hard to breathe/hot/stuffy, exam season (relevant to year 13)  “*I think, potentially in the summer it would be more difficult just because you know it’s warmer and potentially, you know, harder to breathe in a mask if it’s in a stuffy classroom. Um I also think that if it’s during exam season or, you know, like something like that, then pupils minds are definitely elsewhere. And, um, they’re probably going to be less compliant with the trial at those times and that will probably vary uh, from school to school as to when that would be.”* (Teacher)  *“…But yeah, 12 would probably be better and year group to do and it because they don’t do AS anymore, so it would be, you know, in between GCSE and A level, so they would be probably the better year group to do it with I suppose”* (Teacher)  Expert discussion meeting  Population  “Year 12 and 13 (16-18 year old) can consent themselves but not always on site for requited period”  “Year 11 – mindful of exam timetable and last term with friends before moving on “  “Year 7 – new to school and making new friend groups “  “Year 8/9 – more complicated, to be discussed with individual schools“(Experts) | Methodology | Of relevance to recruitment strategy | Ideal population would be:  (a) not an exam year; (b) but also at school full-time. This is to ensure that mask wearing coincides with where their greatest risk of exposure is likely to be.  Ideal year group: year 12 and year 13 |
| 7 | **Barrier: Unclear about what the study involves and risks of taking part**  PPI focus groups  Students takin part in the trial –> Barriers –> Would like to know if there are any risks in taking part  “*Is there any risk*?” (Young people)  PPI focus groups  Other issues considered –> Making sure students understand procedures and what will happen if they aren’t adherent or don’t want to take part  “*Communication, yeah, I think probably a presentation-y thing and then yeah, what happens if they don't want to take part or maybe giving them options. Yeah*” (Teacher)  PPI focus groups  Saliva samples –> Barriers –> Would need to make it clear that we wouldn’t be testing for anything else e.g. drugs  “*I don't know if drug testing pops up in there… No, they they definitely have to make that clear. There would definitely be a few people that would just not go anywhere near that.*” (Young people) | Intervention components | Social cognitive theory would suggest to support self-efficacy for participation by addressing concerns directly (e.g., we can’t/won’t be testing for drug use; masks can be uncomfortable).  Self-determination theory would suggest to provide sufficient information for people to make a choice in order to support autonomous motivation. | The research team would communicate the downsides of participating in the research with students and parents clearly and transparently, making sure they understand the research and had the opportunity to ask questions so that they can make an informed choice of whether to take part (Letters and Q&A sessions with pupils and parents). |
| 8 | **Barrier: Trial stage being too short or too long**  PPI focus groups  6-week time frame –> 6 weeks seems fine  “*I feel like if it was a bit shorter, it might make a little bit more like there’s not really a point but I think feel like six weeks is a good time*” (Young people)  *“I think the 6 weeks is OK*” (Young people) | Methodology | Of relevance to trial design | **Key feature of trial:**  Duration of mask wearing: 6 weeks. |
| 9 | **Facilitator: rules around mask wearing are easy to remember and follow**  PPI focus groups  Facilitators to face masks –> making the rules clear  “*It was really easy for the pupils to remember that they only time they like that they wouldn’t be wearing their masks was wither when they’re outside or when they were eating at lunchtime. That was it*” (Teacher) | Intervention components | Social cognitive theory would suggest to support self-efficacy | **Key features of trial:**  a) The instructions for mask-wearing would be made clear and easy to remember:  i) pupils in the face mask (intervention) group would be asked to wear a face mask when indoors at school, apart from eating and drinking or if they had medical exemptions  ii) pupils in the school rules (control) group would be asked to follow the school’s guidance on face masks at the time during the trial  b) Posters on how to wear masks would be developed, with PPI members. |
| 10 | **Facilitator/Study design: Supply or new/fresh masks**  PPI focus groups  Other issues to consider –> Will we be supplying masks?  “…*you’d have to provide a lot of masks, and it’s how you provide them as well….It would need to be there’s a fresh set of masks at every lesson*” (Parent)  PPI focus groups  Barriers to face masks –>wearing masks properly –> not wearing a clean mask each time  “…*you could be using the same mask for the last, however long, and bring that to school*” (Parent)  “…*I’m sure that sometimes my children just pick up a mask and it’s not theirs*” (Parent) | Intervention components | Social cognitive theory would suggest that the environment should support behaviour change. | **Key features of trial:**  a) Fresh masks would be supplied to all pupils and teachers twice daily  b) Choice of masks: surgical masks  c) Replacement masks to be provided to enable pupils to change masks after each break in the day |
| 11 | **Barrier: High frequency of saliva samples**  PPI focus groups  Saliva samples –> Frequency  “*I feel like once a week is probably the best…anymore than that kind of comes a bit inconvenient*” (Young people)  “*It shouldn’t be more than twice a week*” (Young people) | Intervention components | Social cognitive theory would suggest to support self-efficacy to perform the behaviour by making it sufficiently easy or convenient. | **Key feature of trial:**  Saliva sample would be provided by pupils once a week for duration of trial. |
| 12 | **Study design: Students to report mask wearing behaviours/adherence**  PPI focus groups  Measuring adherence –> Potential methods –> student self-reporting using a book or app  “…*self-reporting that kind of came to my mind…do you give them a book that they’re gonna lose which yeah, and then you could develop an app, but most schools, I mean at our school they’re not allowed phones…*”(Parent)  PPI focus groups  Measuring compliance –> Potential methods –> student self-reporting using online diary/form  *“…I guess you could probably get them to remember, it’s whatever they fill in the diary. But I get enough prompts that I’m then online and filling it, but I do think something that doesn’t get lost as such if you go for the self-reporting*” (Parent) | Methodology | Of relevance to trial design  a) The measure of mask-wearing behaviours would ideally be a combination of self-report by pupils and teachers reporting.  b) The research team would work with the school to identify most acceptable way to measure adherence by teachers.  c) The research team would work with the school to confirm that pupils could self-report weekly as part of the research visit | **Key features of trial:**  a) Self-report of mask wearing by both pupils (weekly) and teachers.  Pupils: Weekly estimate through self-report survey:  How often over the past week did you wear a face covering while indoors at school (excluding times you were eating or drinking)?  - None (0%)  - Up to 25% of the time (e.g., all day but only on one day or for some mornings or some afternoons but not all)  - Between 25% and 50% of the time (e.g., half of the day on most days)  - Between 50% and 75% of the time (e.g., at least half of the week)  - Between 75% and 100% of time (e.g., almost all week)  - Always (100%)  - N/A - I’m medically exempt  Teachers: Estimated percentage of students wearing masks during each class:  - None (0%)  - Up to 25% of pupils  - Between 25% and 50% of pupils  - Between 50% and 75% of pupils  - Between 75% and 100% of pupils  - All (100%) of pupils  After discussion with teachers at the school, a shared Excel file was created for teachers to report the estimated percentages. This form was also available in printed format. |
| 13 | **Barrier: Teachers unable to report/monitor mask wearing behaviours/adherence (Staff time and availability)**  PPI focus groups  School taking part/engagement of staff –> Barriers –> Staff are already under immense pressure  *“…if there’s like they also need to be kind of monitoring and incentivising and um, encouraging pupils in terms of a trial, uhm, that might also be, you know, like it’s just an added thing that needs to be kind of dealt with in policed and etc around the school*.” (Teacher)  PPI focus groups  School taking part/engagement of staff –> Barriers –> teachers are tired and don’t have a lot of spare time-need to not add to their workload  *“I think it needs to be sensitive in terms of the the other things they've got going on. They've got a world of other things going on in your an extra thing and and if you're not careful, yeah you don't want to add to their workload.”* (Parent) | Intervention components | Social cognitive theory and self-determination theory would suggest to build self-efficacy (i.e., competence) of school staff to be able to support the trial procedures. | **Key features of trial:**  See 12  The intervention would minimise pressure on staff by:  a) Making it easy to remember procedure by only conducting the trial within one year group  b) Making completion of data collection (e.g. mask wearing) extremely simple.  c) Going into schools to talk in assembly to support staff to talk about the study.  d) Providing the school with incentives to cover staff time spent on the study. |
| 14 | **Facilitator: Wanting to know whether masks work. Young people would like to know whether masks work and are they necessary instead of simply doing what they have been told.**  PPI focus groups  Students taking part in the trial –> Facilitators –> Gaining knowledge about face masks  *“A desire to ‘find out if they really work’ was prominent among the factors that would encourage them to take part, as well as being part of the research which could answer this question”* (Pre-funding group)  PPI focus groups  Schools taking part/engagement of staff –> Facilitators –> wanting to know if masks are effective  *“…many of us are just following Public Health England as well rather than actually knowing whether it’s effective or not. So you know, that would help get people thinking as well*” (Teacher)  Expert discussion meeting  “Children want to understand why they are being told to do something, even at very young age” (Experts) | Intervention components | Self-determination theory would suggest to build autonomous motivation for participation by drawing on young peoples’ values (of fairness and social justice). | The rationale for the study for pupils should include the social justice considerations – things should be fair (e.g., if we were to ask pupils to wear masks to protect others, we should know if they work and how well they work). |
| 15 | **Facilitator: Consider needs of schools and young people**  Expert discussion meeting  Pre-trial Rational  *“Schools and young people’s needs have not previously been considered*” (Experts) | Intervention components | Self-determination theory would suggest to build relatedness through demonstrating empathy and understanding. | Emphasis in the rationale for the study that schools and young people have not been well-served in the pandemic which means we did not have a strong evidence base of what was effective in protecting young people. |
| 16 | **Facilitator: Enjoyment of being part of research**  PPI focus groups  Students takin part in the trial -> Facilitators –> Enjoying experiments/being part of research  *“I love experimental things*” (Young people)  “*Maybe some of the science, sixth form might be keen to join in just for the experience and you know being involved in a trial would probably be quite interesting to the ones who’ve chosen a levels, yeah, science*” (Teacher) | Intervention components | Self-determination theory would suggest to build autonomous motivation based on opportunities which are valued by young people and schools. | Emphasise in the rationale for the study that the study is an opportunity to engage with research and scientists to both young people and schools. |
| 17 | **Facilitator: Being able to contribute to the society**  PPI focus groups  Students takin part in the trial –> Facilitators –> Frame towards their interests  “*As a generation they are quite kind of aware of social responsibility, sustainability issues, all of that kind of thing….”* (Parent)  “*I guess starting [?] though for helping others… Yeah, and also the benefits of it. .... That increases the lifespan of people and you're protecting others around you too so. Its benefits*.” (Young people)  PPI focus groups  Students takin part in the trial –> Facilitators –> Gaining knowledge about face masks  “*I think it’s for basic awareness, so being part of it is, …, I would be making aware to others*” (Young people) | Intervention components | Self-determination theory would suggest to build autonomous motivation based on constructs which are valued by young people and schools. | Emphasise in the rationale for the study that it is an opportunity for young people to influence the science affecting them. |
| 18 | **Facilitator: Being in touch with higher education**  PPI focus groups  Students takin part in the trial –> Facilitators –> Learning more about universities/talking to people from universities  “…*my children are gonna perking up a little bit when they hear about universities, or if they meet somebody who works at a university… so I don't know whether they might quite like the fact that a university or asking them to do something quite grown up.”* (Parent)  PPI focus groups  Schools taking part/engagement of staff –> Facilitators –> May be a way to get students interested in higher education  “*I think the idea that the pupils will get to experience some sort of higher education experience like research experience or investigation experience from it…would probably be a swayer in terms of senior management being on board”* (Teacher)  *“…having something involved with the school where they can see, you know opportunities post school in an academic setting would be really useful and that would help sell it to them I’d imagine*” (Teacher) | Intervention components | Self-determination theory would suggest to build autonomous motivation based on constructs which are valued by young people and schools. | This study would be promoted as an opportunity to engage with research and scientists to both young people and schools. |
| 19 | **Facilitator: Having one year group. A certain number of staff might already be overseeing the pupils.**  PPI focus groups  School taking part/engagement of staff –> Barriers –> Staff are already under immense pressure  “*I think already having it within one particular year group is probably a really good start, because then you've got a certain number of staff already there are going to be kind of overseeing it… ”* (Teacher) | Intervention components | Social cognitive theory and self-determination theory would suggest to build self-efficacy (i.e., competence) of school staff to be able to support the trial procedures. | See 13 |
| 20 | **Facilitator: Approach head teachers and leadership group to advertise the study.**  PPI focus groups  Advertising of study –> who to aim it at  *“I feel like you should not really, advertise it to the students at all. Kind of advertise it to the uhm, the head teachers and the senior leadership team and make them, like gauge if they’re like kind of up for it…and then they can bring it and talk about it in assemblies or whatever*” (Young people)  “…*when you’ve reached out to the head teachers just see how active they really are…just finding someone like my head teacher that is involved and does make things happen rather than just saying about them happening*” (Young people) | Methodology | Of relevance to trial design | Before the trial starts, the research group would have informal discussions with head teacher and school leadership group to: a) gauge interest b) decide which year group most suitable c) decide on the best methods to approach parents and pupils d) understand what support school would need to participate |
| 21 | **Target behaviour: mask wearing** |  |  |  |
| 22 | **Barrier: Lack of information of the rationale for wearing face masks**  Secondary analysis  Facemask usage-> Impact of time/change in use and attitudes over time  *“I don’t really understand why, …, it just seems a bit silly that they didn’t introduce these measures when it was worse. Like, I don’t really understand.”*  PPI focus groups  Barriers to face masks -> Social influences –> parents attitudes : teenagers may want to rebel  *“I don’t think I’d have any sway. In fact, if I said do it they probably wouldn’t just ‘cause I’d said to do it”* (Parent) | Intervention components | Self-determination theory would suggest to build autonomy to build internal motivation. | The research team would explain the rationale for engagement with the trial procedures in a way that is meaningful to young people, as young people are especially sensitive to external pressure. |
| 23 | **Barrier: Young people feel society has not prioritised them and has in some cases blamed or scapegoated them for aspects of the pandemic. This could create an unwillingness to adhere to societal norms.**  Secondary analysis  Identity-> Young people are blamed  *“I hate that the young people are being scapegoated for the rise in cases, when they literally incentivised to go out and eat”*  *“…they’re urging us to go out, but I know it’s already been said, but it’s like they’re like blaming us all of the time for, you know, going out too much”*  Secondary analysis:  Identity-> Young people aren’t a priority  *“I just don’t think we’re really the most important at the moment, …, we’re at school, and we don’t have exams, like we’re not really the priority”* (Young people) |  | Self-determination theory would suggest to build relatedness through demonstrating respect, empathy and understanding.  Self-determination theory would also suggest to build autonomy to build internal motivation to engage. | The rationale for the study would draw on a social justice arguments to show that young peoples’ and schools’ needs should be considered.  The research team would explain the rationale for engagement with the trial procedures in a way that is meaningful to young people, as young people are especially sensitive to external pressure. |
| 24 | **Barrier: Social context**  Secondary analysis  Face mask usage -> Impact of context  “We will bring masks and we do wear them when we were, like, in the queues and stuff, and cos obviously you have to. But then at, like, Thorpe Park there’s barely any social distancing going on with, like, everyone there. But like in the cars, yeah, we don’t really wear masks”  PPI focus groups  Barriers to face masks –> Social influences -> Students not seeing a lot of mask wearing in other environments  “I think a lot of them, when they're not at school, they don't see a lot of mask wearing. So if you go into shops, there's not necessarily a lot of people wearing masks on public transports, even though they're supposed to. There's it's not necessarily challenged out in.” (Teacher)  PPI focus groups  Barriers to face masks –> Social influences -> Not wanting to stand out  “Fewer and fewer children were wearing face masks and at that point then they didn't necessarily want to kind of be standing out or be different to other people” (Parent) | Intervention components | Self-determination theory would also suggest to build autonomy to build internal motivation to engage. | See 14 |
| 25 | **Barrier: Discomfort**  PPI focus groups  Barriers to face masks –> Practical issues–> Find it uncomfortable  “*It's, it's wearing it for a long time isn’t something which is very comforting”* (Young people)  PPI focus groups  Barriers to face masks –> Practical issues–> Finding it hard to breathe  “…*I got one that wasn't one of the normal, just blue ones it was like um, I don’t know, it was like this, like a quilt one. It was like one that was like specially made it, it just really wasn't that good. And that really kind of put me off. And I found it quite difficult to breathe right the start as well, not because of the the face masks that we have now, like the standard ones, it was just because of the the quilt one that I had.”* (Young people)  PPI focus groups  Barriers to face masks –> Practical issues–> Finding it unpleasant when for more than a couple of hours  “*Sometimes I have to wear it for more than two hours, and that isn't pleasant.”* (Young people)  PPI focus groups  Barriers to face masks –> Practical issues–> Masks make communication difficult for those with hearing difficulties  “*he's my son got significant hearing loss and he uses lip reading and he said to me… he said it protects my teacher, he recognized, it protected his teacher, but he was like I don't know what I'm going to do if my teacher wears a mask”* (Parent) | Intervention components | Social cognitive theory would suggest to support self-efficacy.  Self-determination theory would also suggest to build autonomy to build internal motivation to engage despite barriers. | This study would acknowledge the difficulties of wearing masks (i.e., showing understanding of the issues). Suggestions would be given to resolve these issues (e.g., pupils would be allowed to have short breaks from wearing a face mask if they experience discomfort).  The recruitment would focus on the positive side of the trial.  **Key features of trial:**  See 9 and 10 |
| 26 | **Barrier: Inconvenience**  PPI focus groups  Barriers to face masks –> Practical issues–> Making glasses steam up  “*Uh, people with glasses don't really like it. 'cause it goes up and steams like glasses.”* (Young people)  PPI focus groups  Barriers to face masks –> Practical issues–> Masks get in the way of make up  “*one of mine doesn't like to wear it when she's got a lot of makeup on because it'll wipes off and so when you take it off, you have half makeup, half not, so, so that does come into it”* (Parent)  “*I think lots of the girls, it's 'cause it messes up their makeup. That's what I've heard quite frequently*.” (Teacher) | Intervention components | Social cognitive theory would suggest to support self-efficacy.  Self-determination theory would also suggest to build autonomy to build internal motivation to engage despite barriers. | See 25 |
| 27 | **Barrier: Forgetting**  PPI focus groups  Barriers to face masks –> practical issues –> forgetting  “*sometimes I forget to carry it around*” (Young people)  “*The receptionist said to me the other day that she reckons that we go through about two or three box a day of pupils who don’t bring their own*” (Teacher) | Intervention components | Social cognitive theory would suggest to alter the environment to support behaviour change. | See 10 |
| 28 | **Facilitator:** **Teachers wearing masks (solidarity)**  PPI focus groups  Facilitators to face masks –> Social influences –> Feeling that students assume teachers are doing what they are supposed to - modelling behaviour  “They are quite good at assuming teachers are doing stuff what they’re supposed to do in the students having to do what they’re supposed to do” (Teacher)  Expert discussion meeting  “Teachers in contact with target year group(s) also wear masks – encourage consistency and idea of all being in it together” (Expert) | Intervention components | Self-determination theory would suggest to build relatedness (i.e., a sense that we are all in this together) to support behaviour change. | **Key feature of trial:**  Teachers of face mask (intervention) group would be asked to wear face masks when teaching this year group. |
| 29 | **Facilitator: Peer influences**  PPI focus groups  Facilitators to face masks –> Social influences –> Being part of a group  “*While ‘no-one likes them’, they would wear them if they did so as part of a group*” (Pre-funding group)  PPI focus groups  Student taking part in the trial –> Facilitators –> Influences of friends  “*We make same like decisions*” (Young people)  “*I think whilst us encouraging them to participate in something is is important and helpful, um I think the peer pressure would probably have more of an impact than the parental pressure*.” (Parent)  “*But I think generally for most children, they were, that it would go with what their peers are doing more*.” (Parent)  “*Teachers and parents views were considered important, but the views of their peers were seen as more likely to influence their decisions*” (Pre-funding group) | Intervention components | Self-determination theory would suggest to build relatedness to support behaviour change.  Theory of planned behaviour would suggest to consider the effects of social norms on behaviour change. | Although the trial would only involve two year groups, the whole school would be made aware of the research to build peer support for the year groups involved. |
| 30 | **Facilitator: Having reminders**  PPI focus groups  Other issues to consider –> Are teachers going to be involved in asking students to wear masks? Or other types of reminders?  “*I suppose it's like to what extent are the teachers involved? Are they going to say to everybody as they walk in the room, mask on, mask on, mask on mask on? Or would you, would it be, I don't know, how would they be reminded to do it? 'Cause my feeling is they'd need a lot of reminders*.” (Parent) | Intervention components | Social cognitive theory would suggest to introduce environmental reminders to should support behaviour change. | **Key features of trial:**  a) Poster reminders would be put up in the school  b) See 10 |
| 31 | **Facilitator: fitting the study into existing school activities/making the behaviour easier**  PPI focus groups  School taking part/engagement of staff –> Barriers –> Staff are already under immense pressure  *“…maybe a timetable, Sorry it like a timetable so people know what to expect of them when. Yeah, or like a weekly update or round up or something like that just so it’s not too work heavy on the teachers that you said about the time. And yes, so clear tasks that are required every week would make it easier I think, and you know, really structured*” (Teacher)  Expert discussion meeting  “Build timetable into lessons (science) – provide sample, fill in survey, receive feedback.” (Experts)  “This becomes part of intervention – do in control group too to maintain engagement” (Experts)  PPI focus groups  School taking part/engagement of staff –> Barriers –> Staff are already under immense pressure  *“…maybe a timetable, Sorry it like a timetable so people know what to expect of them when. Yeah, or like a weekly update or round up or something like that just so it’s not too work heavy on the teachers that you said about the time. And yes, so clear tasks that are required every week would make it easier I think, and you know, really structured*” (Teacher) | Intervention components | Social cognitive theory would suggest to introduce environmental reminders to should support behaviour change. | **Key features of trial:**  The trial would be built into the weekly personal development session at school. This could be opportunity to: a) collect pupils’ self-report mask wearing (survey)  b) collect pupils’ report on any adverse events (survey) c) collect pupils’ self-reported mood (survey)  d) collect saliva samples  e) give feedback to the pupils and teachers on progress in previous week  f) let pupils interact with experts |
| 32 | **Facilitator: Getting feedback**  PPI focus groups  Encouraging mask wearing –> Other ideas –> A leader board to track weekly progress  “*Yeah, I think something like a leader board*.” (Teacher)  Expert discussion meeting  “Feedback tailored to each group – reinforce correct elements for control group” (Experts) | Intervention components | Social cognitive theory would suggest to introduce environmental reminders to should support behaviour change. | **Key feature of trial**:  Feedback on the trial progress in the previous week (e.g., the number of pupils taking part, the percentage of pupils wearing face masks, self-reported mood, etc) would be summarised and reported to the pupils and teachers during the weekly research visit. |
| 33 | **Facilitator: Incentives**  PPI focus groups  Encouraging mask wearing –> Incentives  “…*if we are being offered incentive will be good*” (Young people)  “*I definitely think that some sort of like incentive in terms of like, I don’t know voucher or cinema vouchers or something like that*” (Teacher)  “*One other thing that I wonder is whether if you had a slightly bigger group, but you could somehow reward that group if they get a whole week of reporting and of wearing it….some kind of incentive that they’re completing*…” (Parent)  Expert discussion meeting  ->Incentives  “Not necessary with sufficient “buy in”  “Incentives can be barrier as well as facilitator”  “Incentivise school – discuss their preference; reimburse for staff time  “Plaques/photos for future publicity/OFSTED support” (Experts) | Intervention components | Social cognitive theory suggests that manipulation of outcome expectation might promote completion of the behaviour.  Social cognitive theory would also suggest to boost self-efficacy for participation. | **Key feature of trial**:  After discussion with school about incentives, it was decided that:  a) pupils would not be incentivised individually for taking part in the trial;  b) a certificate for participation will be given to the school at the end of the trial;  c) teachers who participated in the study would be incentivised to reimburse staff time spent on the study. |
| 34 | **Target behaviour: providing saliva samples** |  |  |  |
| 35 | **Barrier: In front of other people**  PPI focus groups  Saliva samples –> Barriers –> Might be difficult to do in front of other people  “*It might be difficult*” (Young people)  “*I’m more on the reverse end”* (Young people) |  | Of relevance to trial design | **Key feature of trial**:  Saliva samples would be collected at school during the weekly research visit. |
| 36 | **Barrier: Unclear what the samples are for and how to collect the samples**  PPI focus groups  Saliva samples –> Barriers –> Would need to make it clear that we wouldn’t be testing for anything else e.g. drugs  “*I don't know if drug testing pops up in there… No, they they definitely have to make that clear. There would definitely be a few people that would just not go anywhere near that.*” (Young people)  PPI focus groups  Saliva samples –> Information  “*Um, how many like general spits will it take? Is it like two, is it one? But that’d be about it really. See, I feel like if I was like trying to spit into the tube like three times or four times, it will start to get a little bit, not so easy*.” (Young people) | Intervention components | Social cognitive theory would suggest to maximise self-efficacy by making procedure feasible. | The research team would state clearly in the letters to parent and pupils, information sheet, and consent form that the collection of saliva samples for this pilot study only aims to test the feasibility of weekly collection of saliva samples in schools. The saliva samples collected would only be counted and then discarded, and would not be analysed.  This will also be addressed at any meeting or Q&A sessions with parents and pupils. |
| 37 | **Barrier: Too frequent**  PPI focus groups  Saliva samples –> Frequency  “I *feel like once I feel like once a week is probably the best that would happen, I don't know anymore than that kind of comes a bit inconvenient.”* (Young people)  “*It shouldn't be more than twice a week”* (Young people) | Intervention components | Social cognitive theory would suggest to maximise self-efficacy by making procedure feasible. | **Key features of trial:**  a) Saliva samples would be collected weekly for 6 weeks  b) See 35 |
| 38 | **Facilitator: School having a system in place/Done at school**  PPI focus groups  Saliva samples –> Facilitators –> Some colleges might already have systems in place for lateral flow testing in college so could use similar approach/join onto that  *“...at the minute when we go back in January uh, we go in a day earlier. We have like slots for like our name so it's like A to E, E to H, I to Z, to like go in at certain times to take um like lateral flows. So I feel like you could just do the same system. So get a certain amount people in it for like half an hour.”* (Young people)  PPI focus groups  Saliva samples –> Facilitators –> done at school  “*Compliance would be greatest if samples were collected at school as part of the school day*” (Pre-funding group)  PPI focus groups  Saliva samples –> Facilitators –> done at school  “*Compliance would be greatest if samples were collected at school as part of the school day*” (Pre-funding group) | Methodology | Social cognitive theory would suggest to maximise self-efficacy by making procedure feasible. | **Key features of trial:**  The weekly research visit would take place at the personal development session every Tuesday morning. Pupils from the selected year groups would be asked to attend. For those who took part in the study, they would be asked to complete a self-report survey, and provide saliva samples during this session (data collection). One expert from the research team would be invited to interact with the pupils after the data collection, at the research visit. |
| 39 | **Facilitator: Having reminders**  PPI focus groups  Saliva samples –> Reminders  *“ I could remember if I wanted to”* (Young people)  “*Remembering shouldn’t be hard. Or you could set reminders*” (Young people)  “*I feel like that would help it like um, ‘cause it gets posted over teams everywhere…”* (Young people) | Intervention components | Social cognitive theory would suggest to maximise self-efficacy by making procedure feasible. | **Key features of trial:**  a) Poster showing how to wear face masks appropriately and how to provide a sample sample would be put up in the school.  Have posters at school as reminders.  b) During each weekly research visit, pupils will be reminded to continue wearing face masks and providing saliva samples. |
| 40 | **Facilitator: Having more information/instructions on how to take the saliva samples**  PPI focus groups  Saliva samples –> Information  “*Um, how many like general spits will it take? Is it like two, is it one? But that’d be about it really. See, I feel like if I was like trying to spit into the tube like three times or four times, it will start to get a little bit, not so easy*.” (Young people) | Intervention components | Social cognitive theory would suggest to maximise self-efficacy by making procedure feasible. | **Key features of trial:**  a) Clear and simply instructions on how to provide a saliva sample would be provided to pupils in written format and explained verbally.  b) Posters showing how to provide a saliva sample would be developed, with PPI members. |

### Table B2. Summary of PPI focus group findings

| **Topic** | **Sub-topic** | **Finding** | **Quote** | **Group** |
| --- | --- | --- | --- | --- |
| Barriers to face masks | Practical issues | Forgetting | Sometimes I forget to carry it around | Young people |
|  |  |  | Just like they forget their pencil or they forget their calculator. They forget their mask, they forget their bus pass. | Parents |
|  |  |  | But there are some who definitely have the mindset of you know, like they can't, they find it hard to breathe through them or they you know it's a hassle to carry them around the school and things like that. And they're the ones who tend to to kind of kick up at little bit more of a fuss about it. | Teachers |
|  |  |  | The receptionist said to me the other day that she reckons that we go through about two or three boxes a day of pupils who don't bring their own. | Teachers |
|  |  | Finding it uncomfortable | It's, it's wearing it for a long time isn’t something which is very comforting | Young people |
|  |  | Making glasses steam up | Uh, people with glasses don't really like it. 'cause it goes up and steams like glasses. | Young people |
|  |  | Finding it hard to breathe | Well I have a bit difficulty breathing. And I guess the face mask restricts that whole lot.  I didn't really like it at the start, but that's because I got one that wasn't one of the normal, just blue ones it was like um, I don’t know, it was like this, like a quilt one. It was like one that was like specially made it, it just really wasn't that good. And that really kind of put me off. And I found it quite difficult to breathe right the start as well, not because of the the face masks that we have now, like the standard ones, it was just because of the the quilt one that I had. | Young people |
|  |  |  | But there are some who definitely have the mindset of you know, like they can't, they find it hard to breathe through them or they you know it's a hassle to carry them around the school and things like that. And they're the ones who tend to to kind of kick up at little bit more of a fuss about it. | Teachers |
|  |  | Finding it unpleasant when for more than a couple of hours | Sometimes I have to wear it for more than two hours, and that isn't pleasant | Young people |
|  |  | Masks make communication difficult for those with hearing difficulties | he's my son got significant hearing loss and he uses lip reading and he said to me… he said it protects my teacher, he recognized, it protected his teacher, but he was like I don't know what I'm going to do if my teacher wears a mask | Parents |
|  |  | Masks get in the way of make up | And then the other thing, one of mine doesn't like to wear it when she's got a lot of makeup on because it'll wipes off and so when you take it off, you have half makeup, half not, so, so that does come into it. | Parents |
|  |  |  | I think lots of the girls, it's 'cause it makes up their makeup. That's what I've heard quite frequently. | Teachers |
|  | Changes over time | Getting fed up with wearing masks | I could cope though but I got fed up at an extent | Young people |
|  |  | Feeling that students are fed up with COVID and masks | The majority have maybe kind of become increasingly Uhm, kind of fed up with face masks. | Teachers |
|  |  | Over time teenagers have got a bit fed up/apathetic | I think over time that's changed, so I think they've got to a point where kind of they started to get a bit fed up of it and it became something where less and less fewer and fewer children were wearing face masks.  And then yeah, I think it became a bit apathetic over time. | Parents |
|  | Impact of vaccines | Introduction of vaccines made them feel safer without masks | Now I'm a little bit more I am more confident you know in what I do and then sometimes you know I just go out forgetting the face mask.  Uh, it didn't make me go, oh I'm vaccinated I won't bother. Uh, it did make me feel a little bit more invincible, but I did still wear the face masks. | Young people |
|  |  |  | I do think that the ones who are vaccinated kind of feel a little bit more, um, indestructible | Teachers |
|  | Perception of importance of masks | Masks may be seen as less important when they’re not mandatory | I don't think it’s, I don't take it as important as I did before now. I've actually, you know, reduced the way I used it. | Young people |
|  |  | Students might not see any difference to them when wearing/not wearing masks | It's because situation is been really, being told things are changing, and then they're not really seeing any change. I think for them it's been a very static situation for a very long time, and I think perhaps they're getting a bit bored, probably, as good a word as any. Yeah, you know they're not seeing any difference wearing a mask and not wearing a mask, so they're probably a bit like “well I don't know what the point is anymore”. | Teachers |
|  | Social influences | Students not seeing a lot of mask wearing in other environments | I think a lot of them, when they're not at school, they don't see a lot of mask wearing. So if you go into shops, there's not necessarily a lot of people wearing masks on public transports, even though they're supposed to. There's it's not necessarily challenged out in. | Teachers |
|  |  | Feeling that attitudes at home might influence attitudes to masks in schools | An I wonder whether part of that is from home, so I wonder whether you know they're coming from households in the morning that maybe aren't that bothered about wearing face masks or feel like fed up with COVID or whatever. | Teachers |
|  |  | Influence of teachers behaviours/attitudes | I have to say it there are like teachers who definitely don't wear their masks. Um and also like in between like teachers who do wear their masks but don't challenge pupils who aren't wearing their masks  They're they're quite good at assuming teachers are doing stuff, what they're supposed to do in the students have to do what they're supposed to do.  There's a lack of consistency in mask wearing, like around the school, so they’ll kind of like go to hot spots where they suddenly put them on, um, knowing that it's going to be insisted upon, but in some places I think that it's they get away with it a little bit more. | Teachers |
|  |  | Parents attitudes: teenagers may want to rebel | But, and I think they're still at a stage where they like to think they they kind of want to contradict me, but they don't tend to on those bigger issues that they kind of trust mum on at the moment.  I don't think I'd have any sway. In fact, if I said do it they probably wouldn't just 'cause I'd said to do it | Parents |
|  |  | Not wanting to stand out | Fewer and fewer children were wearing face masks and at that point then they didn't necessarily want to kind of be standing out or be different to other people | Parents |
|  | Beliefs/ attitudes towards COVID | Students who have had COVID but it was mild | Or I don't know whether they're kids. They've had COVID and there are, you know, didn't have it too badly. Uh, consciously or subconsciously, thinking well, it wasn't that bad for me. It's not really that big of a deal. | Teachers |
|  | Impact of government guidelines | Rules keep changing making it hard to keep track of and hard to enforce | Um they keep changing whether we have to wear them or not basically. Um, we're getting it daily updates from Public Health England um telling us what the new guidance is, and also from our local council as well, telling us what to do at any particular time.  The rules have changed quite a few times, so we're really unsure at times whether, as a year group they need to be wearing masks if they're altogether, if they need to be wearing masks um, kind of around the corridors, and that's recently changed, and then the pupils will often use that as a reason for not having their face masks of like, “oh, right, we should be wearing them in the corridors now”, and even though the feeling is is that they probably do actually know that, so because of the the rule changes a lot of the time it's difficult to kind of impose that rule with them | Teachers |
|  | Wearing masks properly | Not wearing a clean mask each time – reusing old masks/other peoples masks | There was a bit of again a bit of badge of honour about if you could be using the same mask for the last, however long, and and bringing that to school, and it was like, “oh X has only ever, uh, changed his mask once all this half term”  I have to be honest as well, I think sometimes they're switching, so I'm sure that sometimes my children just pick up a mask and it's not theirs. | Parents |
|  | Wanting to go back to normal | Not having to wear masks feels a bit more normal | I think the difference between mandatory to then going back so I think there was almost a bit of a celebration of the fact that, oh, great, we're no longer having to go, to wear, to wear masks actually by the school, as much as anything for us. So it was like oh thank goodness school is beginning to turn a bit that back to normal. | Parents |
|  | Mask exemptions | A lot of students with medical exemptions which seems to not be being checked as much, or they have exemptions but were initially wearing masks but not any more | The numbers who are exempt seem to have gone up, but obviously the number of kids with conditions that require them to be exempt hasn't gone up, so it's just who was prepared to wear a mask before and who isn't now essentially. | Teachers |
|  | No one likes them |  | ‘No one likes them’ | Pre funding group |
| Facilitators to face masks | Beliefs about safety and protection | Seen as a way to protect yourself or others from airborne diseases/  Wanting to protect their own health | I feel like it's just really important. I'm not exactly sure what the percentages are for light preventing you from passing anything on to catching it by just. I feel like it's just unnecessary precaution. For your own safety in other peoples.  Masks are mainly for protection against diseases which fly around  You have to protect yourself and you know the best way to do it  So to me it's all about protection  It wasn't difficult at all for me because if it's to do anything for the sake of my health, I'll go the extra mile | Young people |
|  |  | Feeling that students understand it is important, especially when the situation is getting worse due to new variants | So on the whole they they do understand that it you know, like the situation is worsening and that it is important that they wear masks. | Teachers |
|  | Masks are important |  | It's very important as well  I think I'm um the use of face masks is very important. | Young people |
|  | Masks being mandatory | Wearing masks because its mandatory/enforced | At first the reasons for is wearing the face mask was for our own safety because of COVID, but later on it became something more mandatory and also to avoid other various diseases.  Face mask was compulsory for us. We learned to live with it | Young people |
|  |  | If there are punishments for not wearing masks e.g. extra work | Ok It could be you know, staying late or you know you giving them you know something extra you know to work on… OK, I don't think punishment is something which you know anybody want to get it, right from very small or very big so. Like I said before, you know you getting getting extra works, you know or we get getting extra assignment or things for you to work on. | Young people |
|  |  | Making it mandatory takes the thinking/any discussion away and makes it easier for teachers to enforce – also makes it easier when the rules are simple | I think mandatory just because it took the thinking away for them. It took quite a lot for discussion away, and it was, for teachers, it was a lot easier when it's mandatory 'cause it's, you know there's no messing around, you put one on, that's it. And yeah, I think it was easier when it was mandatory.  It was really easy for the pupils to remember that the only time they like that they wouldn't be wearing their masks was either when they're outside or when they were eating at lunchtime. That was it. So in terms of remembering any rules, there was like minimal kind of brain power that was needed for that... when it was just like a blanket rule, it was it just they kind of got into a habit | Teachers |
|  |  | Students will wear masks around teachers they know are strict on it | What I would say is that they have definitely picked up in my school on the teachers who will insist on mask wearing and a really strict on it And those who aren't... so they were kind of like go to hot spots where they suddenly put them on, UM, knowing that it's going to be insisted upon, but in some places I think that it's they get away with it a little bit more | Teachers |
|  | Beliefs about susceptibility/vulnerability to COVID-19 | Having vulnerable family members | And another, any specific situations or factors that might influence how important the masks are?  … Uh, probably just be like a few family members that are quite vulnerable potentially. | Young people |
|  |  | Fear of COVID-19 | And then sometimes they get very distressed and then feel like moving it but um, you know, the um the fear of the virus is what’s making, you know, was making me keep it on 'cause I know you fear of them or not getting the virus so you have to do it.  Whenever I’m out without taking my COVID precaution, I’m always worried and scared. | Young people |
|  |  | Being more vulnerable/needing to shield | We had a few pupils at our school who didn't come in for the whole of lockdown, so they were away for more than a year and when those people came back, they were the ones who took mask wearing very seriously for obvious reasons that they have been shielding for all of that time, and they're they're still the ones today who will, for example, wear masks in class even though it's not insisted upon in our school.  Same, kids have been at home for a very long time have obviously got people who are shielding or themselves or shielding, so you know, sort of taking it very seriously and will still be wearing their masks now. | Teachers |
|  |  | Initially teenagers felt relieved and enthusiastic | Children were very enthusiastic and very kind of relieved in a way. | Parents |
|  |  | Direct experience of COVID | I do wonder what there's an impact of whether they've had COVID or they've had COVID in their family, or they've had some personal experience as well, whether that it, whether suddenly kind of their mask wearing goes up when their awareness goes up. | Parents |
|  | There’s no harm | Feeling that there’s no harm in wearing a mask | I feel like there's no harm in wearing it, even it, even if it made you 5% less likely to catch anything or five cent less likely to pass it on. You know it's still. | Young people |
|  | Impact of the context | Being in a more crowded environment | If they did like become a big crowd of people I have put it on, you know when it was optional. But I’ve always had one in my pocket in case I get into any kind of situation like that. | Young people |
|  | Social influences | Feeling that students assume teachers are doing what they are supposed to – modelling behaviour | They're they're quite good at assuming teachers are doing stuff what they're supposed to do in the students have to do what they're supposed to do. | Teachers |
|  |  | Parents attitudes: teenagers trust parents on big issues like COVID | My children probably think on Mum has probably looked into it a bit more than we have | Parents |
|  |  | Wanting to protect teachers/others | He said it protects my teacher, he recognized, it protected his teacher | Parents |
|  |  | More likely to wear mask if everyone else is | There's a bit of a following thing kind of in terms of if others are doing it then people people will do it | Parents |
|  |  | Being part of a group | While ‘no-one likes them’, they would wear them if they did so as part of a group (rather than individuals) | Pre funding group |
|  | Psychological influences | Can hide behind it when feeling anxious | And then one of them kind of quite liked it in terms of if you're feeling anxious, it feels a bit like you can hide behind it, like like children hide behind their hair. It's a bit the same kind of thing if you have a mask on, actually that can feel a little bit kind of protected from the world. So I think there is an emotional relationship with the mask as well. | Parents |
|  | Appearance | Masks hide spots | Thing is he used to come home and say it was quite enjoying wearing the mask for the fact that it hid all his spots.  One of my children really likes the fact it covered up some of her skin which is quite helpful 'cause then you don't see anybody’s spots which is lovely. | Parents |
|  |  | Having different coloured masks | I didn't realize this but in Italy have every single colour of mask under the sun and so you buy, 'cause you have to wear surgical masks over there and say like they came back with these bright coloured FFP1 masks that they’re very proud to wear and it was kind of yeah it became, it's become a bit of a kind of a thing now as well, so that's been quite interesting for me to watch and, their reactions that my son loved wearing a bright pink one, um 'cause that's what he does. | Parents |
|  | Rationale for mask use |  | While ‘no-one likes them’, they would wear them if the rationale for doing so was of relevance to them | Pre funding group |
|  | Changes over time | Got used to wearing a mask | Well, at first it was a bit difficult, wearing it in school, but I I think I got used to it, so I started wearing it more often and. Yeah, I was used, to it pretty much… So I could, almost part of me now. | Young people |
| **Trial procedures** | | | | |
| Students taking part in the trial | Facilitators | Enjoying experiments/being part of research | I love experimental things | Young people |
|  |  |  | Maybe some of the science um, sixth form might be keen to join in just for the experience and and you know, being involved in a trial would probably be quite interesting to the ones who've chosen a level, yeah, sciences.  I think it would be really important to tell them that it is a trial and the importance of the trial | Teachers |
|  |  | Testing for COVID/other illnesses is seen as a benefit | I think samples would help convince people to participate  I would want to participate… since there is going to be a test for COVID another flu | Young people |
|  |  | Wanting to push for masks to be more common | I'm not sure if this is your what you're trying to push, but trying to push masks to kind of be more common in society. I feel like I I I totally agree with trying to make more people just wear it to where it rather than having to. I'm not sure if that's true agenda, but I feel like that would work for me. | Young people |
|  |  | Influence of friends | We make same like decisions | Young people |
|  |  |  | I think whilst us encouraging them to participate in something is is important and helpful, um I think the peer pressure would probably have more of an impact than the parental pressure.  But I think generally for most children, they were, that it would go with what their peers are doing more. | Parents |
|  |  |  | Teachers and parents views were considered important, but the views of their peers were seen as more likely to influence their decisions | Pre-funding group |
|  |  | Gaining knowledge about face masks | A desire to ‘find out if they really work’ was prominent among the factors that would encourage them to take part, as well as being part of the research which could answer this question. | Pre-funding group |
|  |  |  | I think it's for basic awareness, so being a part of it is, I think it will be, I think it's opportunistic, opportunistic. As I would be making aware to others  Since it's for an educative purpose | Young people |
|  |  | Explaining that they’re being compared to another school | I think it would be really important to tell them that it is a trial and the importance of the trial and the fact that they're being compared to another school, I think that would probably get a lot of pupils on board because of the fact that they see a kind of significance to it. | Teachers |
|  |  | The right kind of incentives | If you offered them a, you know a cost of voucher or something at the for for the class that done at the most at the end of the week, that kind of thing has currency with them. And so I think it's finding some it's appropriate for that age group. | Parents |
|  |  | Frame towards their interests | As a generation they are quite kind of aware of social responsibility, sustainability issues, all of that kind of thing. So I think if you talk to them about the the social good or the potential good that may come from their research, I think that would speak to them, I think, but alongside the short term reward of you can have a Costa in five minutes, there's also that this is generally a good thing to do. | Parents |
|  |  |  | I guess starting [?] though for helping others… Yeah, and also the benefits of it. Are you talking [?] do this, then you have the outcome. It's, your shielded from this disease or this virus and yep. That increases the lifespan of people and you're protecting others around you too so. Its benefits. | Young people |
|  |  | Having researchers visit the schools to talk about the study – particularly someone close to their age. Try to establish a relationship | I think that would be an in person thing with not, they don't like it when it's a massive assembly type thing. I think it would be at class level talking to them and talking to them like an adult. Being honest with them about what it's about and and maybe putting in some little ascent incentives but not making that the whole reason to do it, because they'll feel patronized by that. I think give them the wider thing and then say, but we understand this is an imposition, so we're going to try and help you in this way, but I still think they want the the proper rationale.  And I think the fact that if you've got people who are like them in the sense of closer to their age and less parental age, uh, that that can also help with just kind of yeah, that's a person I can kind of relate to in some way that I don't relate to in the sense that it's going to be my mum type of thing... I think I suppose what we're looking for is some, for them to identify “I could be that person in three years time rather” than that person does the same job as my mom. | Parents |
|  |  |  | You should, someone should like be, should have an insider like to talk to students | Young people |
|  |  | Getting out of lessons as part of the research | Uh, “we can get off lessons on this one because we get join a focus group” or whatever it is, you know that kind of, that's one of the rewards for them. Although it's funny because at the same time they don't like missing school now because they've realized what it's like, but just the odd half an hour out of lesson is just yeah, that in itself is quite reward, isn't that? | Parents |
|  |  | Learning more about universities/talking to people from universities | And that age group are starting to think about what they're going to do at 18, so when, so I've noticed my children are gonna perking up a little bit when they hear about universities, or if they meet somebody who works at a university… so I don't know whether they might quite like the fact that a university or asking them to do something quite grown up. | Parents |
|  | Barriers | Apathy | I guess maybe it's usual that there's apathy in crucial areas. So people wouldn't as for that reason. | Young people |
|  |  | Parental restrictions/consent | I think it would need parental consent  I'd probably take part, but parental awareness is still more needed. | Young people |
|  |  |  | I think you'll have a few parents who will say no anyway, 'cause they're always are some parents who are very wary of any data being collected on their children, and when they are asked the question, err on the side of caution. | Parents |
|  |  | How influential parents are and what their views on masks are – some people might want to rebel | It depends how influential their parents are over them. I mean I don't particularly feel that influenced, but some people might be more, or some people might just want to rebel. I mean at my age a lot of people like to rebel against what their parents think, so it might be a problem. | Young people |
|  |  | Don’t like feeling forced | You don't feel you're being forced to do it | Young people |
|  |  | Influence of friends, likely to do what friends do | We make same like decisions | Young people |
|  |  | Would want to know if there are any risks | Is there any risk? | Young people |
| Schools taking part/ Engagement of staff | Facilitators | May be a way to get students interested in higher education | I think the idea that the pupils will get to experience some sort of higher education experience like research experience or investigation experience from it, like actually kind of be involved in the process and see how it's conducted, that would probably be a swayer in terms of senior management being on board.  I think we don't have a particularly high uptake for university, and I think having something involved with the school where they can see, you know opportunities post school in an academic setting would be really useful and that would help sell it to SLT I'd imagine | Teachers |
|  |  | Explaining importance and rationale of the trial | I think, like with the pupils making it really obvious the purpose of it and the significance of it and why it's being done and why it's important. That definitely kind of increases buy in, um. So just kind of like being transparent on details and and rationale, I think would be really important. | Teachers |
|  |  | Wanting to know if masks are effective | Because you know, many of us are just following Public Health England as well rather than actually knowing whether it's effective or not. So you know, that would help get people thinking as well. | Teachers |
|  |  | Transparency of whats involved | Just kind of like being transparent on details and rationale, I think would be really important | Teachers |
|  |  | Benefit for teachers of students in their class wearing masks | I wonder as well whether there's any any sort of selling of the benefits of the fact that actually you could end up with a class full of mask wearing kids, which I'd imagine it's quite attractive to teachers.  Yeah, I think particularly if it went up to a holiday period. I think teachers would be really relieved if they happen to be in the the the half of the study where they had mask wearing in their class all the way up to the holiday, because so many teachers in the last two weeks of term start to look and say “right I can't see my grandparents for Christmas if, I can't go on holiday to Spain if” so, they're kind of starting to panic a little bit towards the end of term. | Parents |
|  | Barriers | Staff are already under immense pressure, and this would be an added thing to monitor/police/encourage. To overcome this: working collaboratively with staff, providing a structured timetable or a weekly update, or clear weekly tasks so teachers know what is expected of them – things to reduce the workload for teachers | I think the potential issue might be staff time. So staff feel under kind of immense pressure as it is, and if there's like they also need to be kind of monitoring and incentivizing and um, encouraging pupils in terms of a trial, uhm, that might also be, you know, like it's just an added thing that needs to be kind of dealt with in policed and etc around the school.  I think already having it within one particular year group is probably a really good start, because then you've got a certain number of staff already there are going to be kind of overseeing it… I think just having like maybe time allocated to providing little updates or kind of um reminders and things like that, that might just kind of fill up some tutor times or some assemblies, and then it kind of takes that maybe the pressure off the school in other ways to have to be provide, like using up their time basically, so I wonder if it was kind of done it as a um collaboration, like more collaboratively between the staff and the university then that, might help to alleviate some staff pressure.  Uh, maybe a timetable. Sorry it like a timetable so people know what is expected of them when. Yeah, or like a weekly update or round up or something like that, just so it's not too work heavy on the teachers that you said about the time. And yes, so clear tasks that are required every week would make it easier I think, and you know, really structured. | Teachers |
|  |  | Teachers are tired and don’t have a lot of spare time – need to not add to their workload | I think it needs to be sensitive in terms of the the other things they've got going on. They've got a world of other things going on in your an extra thing and and if you're not careful, yeah you don't want to add to their workload. | Parents |
|  |  | Need to make sure it doesn’t come in the way of education needs | And the other thing is obviously there's a lot of concern around students of that age in terms of how well they'll do in their A levels, they didn't do the full GCSE's, um that, it's kind of, people are very worried about the education of that year group, so you just have to be cautious I think about making sure that doesn't feel like it's something that would get in the way of anything like that. | Parents |
| Encouraging mask wearing | Incentives | Incentives e.g. stationary, vouchers, being included in research papers (names or photos), cinema vouchers | Yeah maybe if we are being offered incentive will be good  I feel like an incentive of maybe being included in some things, so I don't know, listing their names or something at the end of a research paper maybe. Like having photos of them published with the paper as well. Maybe another £10 voucher | Young people |
|  |  |  | I definitely think that some sort of like incentive In terms of like, I don't know voucher or cinema vouchers or something like that | Teachers |
|  |  |  | I do think the Costa idea is classic. It's it. They like to feel independent, they like go to a cafe. | Parents |
|  |  | The right kind of incentives – not amazon vouchers, something more instant | I’ve found the voucher is quite an interesting one recently because even Amazon vouchers and stuff just don't seem to be as. No no.  It’s like that instant gratification of whatever it is it's like now. | Parents |
|  |  | Reward/prize if wearing mask for whole week | One other thing that I wondered is whether if you had a slightly bigger group, but you could somehow reward that group if they get a whole week of reporting and of wearing it. And whether that's just points and then it goes into some kind of prize at the end or some kind of incentive that they're competing 'cause, yeah, they they like element of competition and kind of and then they're doing it for each other as well in terms of “oh we can't forget because otherwise I’m going to let the side down”. I suppose it might lead to some dishonesty. | Parents |
|  |  | Punishment rather than incentives | Well, I, I guess I would say for children like you don't [?], you could use it as a form of, oh, you could tell them that theyuse it they'd get probably treat or something sweet candy. But talking about teenagers right now, I don't think that would work. Or probably you just get detention [with that] if you use it then you skip detention, or if you're caught without the nose mask you get detention, probably. | Young people |
|  | Competition | Competitive element could work | I guess you really haven't thought about exactly how you would make it work, but I feel like it would work. | Young people |
|  |  | Using the buddy system as a competition between them | And, uh, competition probably would help as well, so if they're, yeah, they’ve got a friend doing it as well and a buddy, this sort of competing with each other I suppose as well as the other school or whatever. Uh yeah, I think that could appeal to the more competitive students, yeah. | Teachers |
|  |  | Element of competition may be useful | And whether that's just points and then it goes into some kind of prize at the end or some kind of incentive that they're competing 'cause, yeah, they they like element of competition and kind of and then they're doing it for each other as well. | Parents |
|  |  | Mixed views | Views were mixed on this issue, with some thinking it would encourage young people to take part and engage for the 6 week observation period, while others thought it may trivialise the work and make some take it less seriously | Pre-funding group |
|  | Collaboration | Having an overall collaborative goal could work | Overall goal is acceptable  Yeah, I guess it would since the [work?] is now in group, so definitely people from your class would want to participate. So give them their face mask and you get more, you get more people doing that. And the award or reward would be for the class or the whole team. | Young people |
|  |  | Peer pressure/collaboration may backfire if very defiant students influence others | I think there's some where you have where you have really strong defiant personalities uhm, often there's a bit of a ripple effect within a group of pupils around those particular individuals, and I wonder whether it would lead to a whole class kind of not being, being less compliant, potentially as a result. Nobody wants to be the one who's like 'cause the nerdy, the nerdy kid who's like trying to encourage others when they don't want to be. So I think it could almost go both ways potentially. | Teachers |
|  | Mask ‘buddy’ | Using the buddy system as a way of reminding them, or as a competition between them | So the buddy situation would be a bit little bit like the AA. Somebody keeping them on task or whatever, so that would be helpful to keep them going up. | Teachers |
|  |  | Peer buddy system may be difficult due to complex relationships at that age i.e. friends falling out regularly – maybe better if self-selected | I think relationships are quite challenging at that age, so it you'd need an enormous amount of help to work out how to put buddies together, so you'd probably requiring quite a lot from the school in terms of finding the right, unless they chose their own, maybe. I don't know, I think because they, well, maybe this isn't, maybe this is just my children, but because they fall out with other people so regularly and then they make friends again and they fall out again and they change friendship groups. Or because all those things are happening, I think it would be difficult. But if it was self selected it could work I suppose. | Parents |
|  | Other ideas | Different colours for each day | Maybe you need Louise’s beautifully coloured ones. Maybe if they all had a different colour on each day they’d be “oh yes”. You'd be able to see then as well if they got a fresh one. | Parents |
|  |  | Providing facts about COVID – stories about bad cases and from people of the same age | I mean I would say like maybe scared people a little bit, some facts about omnicron… So maybe like a medium like fair arousal type thing, so not like really scary stuff, but not like maybe, maybe try and tell some stories about like bad cases maybe. And like people our age. | Young people |
|  |  | A leader board to track weekly progress | Yeah, I think something like a leader board. | Teachers |
|  | Peer pressure |  | I definitely think that peer on peer pressure works a lot more effectively than teacher on pupil pressure | Teachers |
| Saliva samples | Facilitators | Wanting to find out if positive for anything | I think samples would help convince people to participate | Young people |
|  |  | Some colleges might already have systems in place for lateral flow testing in college so could use similar approach/join onto that | at the minute when we go back in January uh, we go in a day earlier. We have like slots for like our name so it's like A to E, E to H, I to Z, to like go in at certain times to take um like lateral flows. So I feel like you could just do the same system. So get a certain amount people in it for like half an hour. | Young people |
|  |  | Done at school | Compliance would be greatest if samples were collected at school as part of the school day | Pre-funding group |
|  | Barriers | Might be difficult to do in front of other people – would prefer to do it at home | It might be difficult  I’m more on the reserved end | Young people |
|  |  | Would need to make it clear that we wouldn’t be testing for anything else e.g. drugs | I don't know if drug testing pops up in there… No, they they definitely have to make that clear. There would definitely be a few people that would just not go anywhere near that. | Young people |
|  | Reminders | An app reminder might be helpful, others felt they would remember fine by themselves. | I could remember If I wanted to.  Remembering shouldn't be hard. Or you could set reminders | Young people |
|  |  | Reminders on Teams from staff has helped for lateral flow testing. | I feel like *that* would help it like um, 'cause it gets posted over teams everywhere. I mean, I've seen the same thing with them telling us about January twice. I will probably get a thing a week before and then a thing a day before like popping on teams, to come in. | Young people |
|  | Frequency | Some felt once a week was fine, some felt less frequent would be better | I feel like once I feel like once a week is probably the best that would happen, I don't know anymore than that kind of comes a bit inconvenient.  It shouldn't be more than twice a week. | Young people |
|  | Information | Would want to know how long it takes/how much saliva it takes | Um, how many like general spits will it take? Is it like two, is it one? But that’d be about it really. See, I feel like if I was like trying to spit into the tube like three times or four times, it will start to get a little bit, not so easy. | Young people |
| 6-week time frame |  | 6 weeks seems fine | I feel like if it was a bit shorter, it might make a little bit more like there's not really a point, but I think feel like six weeks is a good time.  I think the 6 weeks is OK. | Young people |
|  |  |  | They've been wearing masks now for like over a year and so I think that in terms of a time frame that probably That would probably be fine. | Teachers |
|  |  | Adherence might drop off | Whether you would then get some kind of fall away of compliance in weeks five and six, you might. | Parents |
|  |  | Carving up the time might be effective i.e., three 2-week intervals | Hearing six weeks for for them, though they’ll equate that as half a term. Half a term, it takes forever for half a term to go by, um and and so I do wonder whether they’d be put off by that, and I wonder if there's some way that there could be some kind of, it’s almost selling it to them in a slightly different way about how it's carved up, so that it's three blocks of two weeks or something | Parents |
| Measuring compliance | Potential methods | Select a few students to report adherence at break/lunch times | I feel like you can get a select few students to report themselves. Maybe like if it group of people to this walkabout, break or lunch time. | Young people |
|  |  | Student self-report e.g., using a stopwatch/timer for when they wear it, phone app -> links in to giving them responsibility | Or they could do some sort of timer situation a like a stopwatch-y thing, so, click it on and off. You know that would be reliant on them being honest as well I suppose.  if they had a small timer with them or even something on their phone, if there's an app or something, I don't know how difficult is to run it. But you know something where they, 'cause they’re on their phones all the time, like logging something, there must be some app that measures, time, a clock I think they're called. I don't know. I mean, [something?] that they could, you, you know, enter their own data on that could be centrally collected, or something, might be helpful so they're uploading their own data. Then they have a bit of a sense of responsibility as well about providing accurate data. | Teachers |
|  |  | Taking photographs in classrooms | My main thought is that you can see it, so it's kind of like if you took a photograph, class photograph and it's difficult to get consent for photographs, but if you take class photograph, you've got a sense of how many and where they are sitting, so it gives you a sense of not just who was and wasn't wearing a mask but where they were in terms of where they were clustered together. | Parents |
|  |  | Physically observing classrooms | That's the only thing I can think of into 'cause otherwise you'd be requiring either someone to physically observed that which would be kind of impossible. | Parents |
|  |  | Students self-reporting using a book (but may lose it) or an app (but phones aren’t allowed) | Yeah, it was self reporting that kind of came to my mind first of all and I was like, well, what do you, do you give them a book that they're they're gonna lose which yeah, and and then you could develop an app, but most schools, I mean at our school they're not even allowed phones in in in school let alone, you know not even allowed to take them into school. Uh, although actually in sixth form they are, so yeah that's yeah, but yeah so. | Parents |
|  |  | Students self-reporting using online diary/form | I'm helping out with a trial at the moment on antivirals for COVID and I I'm I'm getting a diet diary each day and I guess you could probably get them to remember, it's whatever they fill in the diary. But I get enough prompts that I'm I'm I am then online and filling it in, but I do think something that doesn't get lost as such if you go for the self reporting. | Parents |
|  | Potential issues/ considerations | Would need to account for number of students actually present each day | You'd have to consider how many people would be in college on that day. Because I've I've got a few days where I'm in for two lessons. On a Tuesday, I’m in for two lessons and then I go home so I won't really be included on that day. On Thursday I’m in for one lesson, and then I go home and other people by not be in at all for one day. So you'd have to like make sure you get the population for that day before you figure that out. | Young people |
|  |  | Could be monitored in classrooms but not in other areas of the school | I think that's really difficult because you're not with somebody all day, so it would be easy to say like in each lesson to monitor it, but actually their movement around the school is completely unmonitored or like it's monitored by different members of staff basically. | Teachers |
|  |  | Teachers are too busy to record adherence themselves | …or you'd be asking the teacher to make a note of how many people that day were wearing a mask, and that's too onerous, they're already busy trying to do 3000 other things | Parents |
| Advertising of study | Who to aim it at | Aim it at head teachers/senior leadership team for them to bring to students | I feel like you should not really, advertise it to the students at all. Kind of advertise it to the uhm, the head teachers and the senior leadership team and make them, like gauge if they're like kind of up for it, and if they're like active within the school. And then they can bring it and talk about it in assemblies or whatever. | Young people |
|  |  | More active teachers (on google classroom/teams) might be more engaged | I feel like maybe, when you've reached out to the head, teachers just see how active they really are. So maybe just take a look at the systems, how active they are on teams or Google Classroom or Show my homework, that’s another one. And just see how active they are… So I feel like um, just finding someone like my head teacher, that is involved and does make things happen rather than just saying about them happening. | Young people |
|  | How to advertise | Social media | Social media like instagram/Tiktok with posts including both researchers and young people | Pre-funding group |
|  |  |  | That's basic social media because we spend more time on Instagram and like well, Twitter, but mostly Instagram and yeah so. Advertising on Instagram would be good. | Young people |
| Other issues to consider |  | Unlikely that a whole year group would take part/wear masks properly | Yeah yeah, I feel like you've run into problems with that. Just with, yeah, I feel like [?] if I said about the year 13's in my college, I'd say there's um, I don’t go to a particularly big college. I think there's about 120 my year and then about 140 year twelves. But I’d probably say at least 10 out of my year would, would not be wearing them all the time and would have to be told to put it back on and then take it off as soon as they walk around the corner. So I feel like that would kind of affect your results. | Young people |
|  |  | Would we be measuring during free periods | Oh, and free periods as well. And just to see people like wearing them in the library. | Young people |
|  |  | Concerns about other influencing factors e.g. exposure outside school/college | I'm not really sure how you be able to like cancel out the other variables, like um bigger families of those people, so they see more people like outside of college. Uh, and I like there would be a lot of outside influence from outside of college. | Young people |
|  |  | What would happen to pupils if not compliant, would there be punishment, what can teachers do – maybe those that aren’t compliant don’t receive incentive | I think that it would be really important to consider like along with the school What will happen to pupils if they aren't compliant… and in that situation, is it going to be like a carrot or a stick approach like will they be punished in any way for having not followed those rules And if so, like what would that look like? | Teachers |
|  |  | Making sure students understand procedures and what will happen if they aren’t compliant or don’t want to take part | I think that it would be really important to consider like along with the school, what will happen to pupils if they aren't compliant… is it going to be like a carrot or a stick approach like will they be punished in any way for having not followed those rules, and if so, like what would that look like?  Communication, yeah, I think probably a presentation-y thing and then yeah, what happens if they don't want to take part or maybe giving them options. Yeah | Teachers |
|  |  | Self-isolation may impact the number of students who are available | And obviously quite a lot of them are off at the moment with COVID, so they're in and out, we we've gotten inconsistent sort of cohort at the moment. There's, the numbers keep changing, so yeah, I think it be numbers mainly. | Teachers |
|  |  | Summer would be more difficult in terms of timing – more complaints about being hard to breathe/hot/stuffy, exam season (for year 13s only) | I think, potentially in the summer it would be more difficult just because you know it's warmer and potentially, you know, harder to breathe in a mask if it's in a stuffy classroom. Um I also think that if it's during exam season or, you know, like something like that, then pupils minds are definitely elsewhere. And, um, they’re probably going to be less compliant with the with the trial at those times and and that will probably vary uh, from school to school as to when that would be.  Yes, summer and exam season sort of tie in as well, though with each other, don't they really? But yeah, 12 would probably be better and year group to do and it because they don't do AS anymore, so it would be, you know, in between GCSE's and a level, so they would be probably the better year group to do it with I suppose. | Teachers |
|  |  | Are teachers going to be involved in asking students to wear masks? Or other types of reminders? | I suppose it's like to what extent are the teachers involved? Are they going to say to everybody as they walk in the room, mask on, mask on, mask on mask on? Or would you, would it be, I don't know, how would they be reminded to do it? 'Cause my feeling is they'd need a lot of reminders. | Parents |
|  |  | Will we be supplying masks? How frequently? | I think you're absolutely right about having to wear, you'd have to provide a lot of masks, and it's how you provide them as well. If you just issued each child with a box of mass on Monday and said there you are for six weeks, you've got no chance. It would need to be there's a fresh set of masks at every lesson. | Parents |
|  |  | Not worth trying to enforce it outside of classrooms –just focus on within classrooms | I wonder if you’re kind of almost setting yourself up to fail by trying to make it all day, and whether you need to do, kind of just accept that maybe morning and afternoon, but I don't know  If it wouldn't ever be possible to enforce wearing them at dinner times, how useful is that in your study? | Parents |
|  |  | Important to assess mask use in control group as well as mask group, as some people choose to wear it when not mandatory | I do remember right at the beginning my son had said to me “oh yeah, now in school if anybody’s got a cold they just automatically wear their masks”. So at the point when they were wear it, when they were doing the only the corridors thing, it was, just became the done thing. And I sense that’s still the case. Um, so if you have got your control group of just following government guidelines whether there will be self imposing some mask wearing if they've got a sniffle or whatever it might be. | Parents |
|  |  | Need to be inclusive to all students and have solutions for potential issues e.g. how to involve those with disabilities/additional needs | I suppose if it was a school where they had a high number of SEN children or something, or students, you would need to um, to be mindful of of that. Uh and yeah, have solutions, whether that be, I mean a lot of people talk about these see through masks, but they're not actually that useful for people who who lip read most of the time for various reasons. So it is just trying to think think about some of those aspects so that it can be an inclusive, it can be inclusive. But at the same time my plea would be not to exclude them just because “oh that's a SEN child”, that, 'cause actually they can probably give some very very useful data and widen that that view. | Parents |

# Supplementary Appendix C

## Example of guidance on the appropriate way to wear a face mask


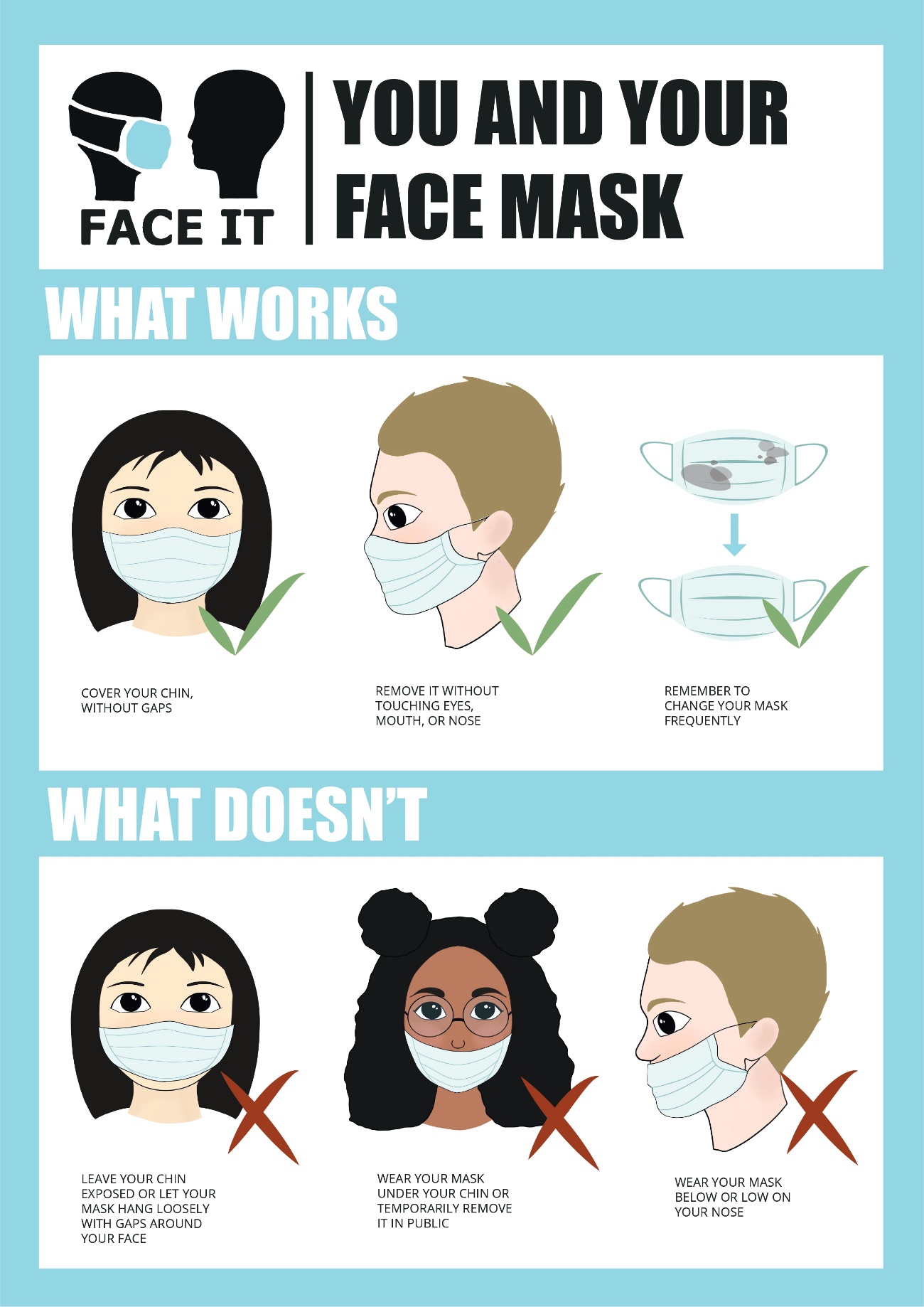


## Example of guidance on how to collect a saliva sample

**
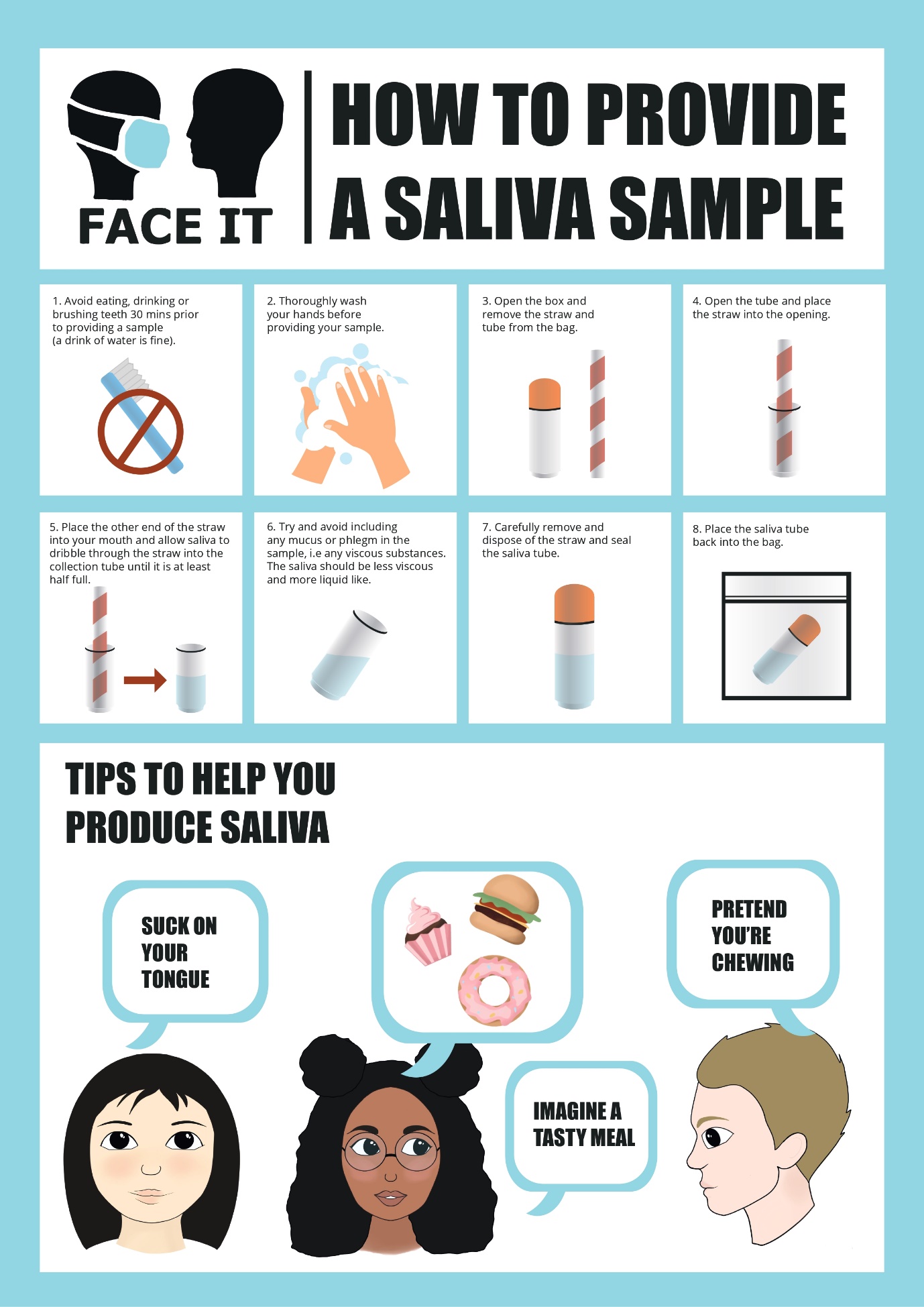
**

## Example of a weekly survey

**Section A**

| 1. Please enter your age (please write down a number): |
| --- |

| 2. How would you describe your gender? | |
| --- | --- |
| Male |  |
| Female |  |
| Non-binary |  |
| Gender-fluid |  |
| Prefer to self-describe: please specify |  |
| Prefer not to say |  |

| 3. How would you describe your ethnic background? | |
| --- | --- |
| White – British, Irish, other |  |
| Asian/Asian British – Indian, Pakistani, Bangladeshi, other |  |
| Black/Black British – Caribbean, African, other |  |
| Chinese/Chinese British |  |
| Mixed race – White and Black/Black British |  |
| Middle Eastern/Middle Eastern British – Arab, Turkish, other |  |
| Mixed race – other |  |
| Other ethnic groups |  |
| Prefer not to say |  |

| 4. Which year are you in?  (please select an answer) | |
| --- | --- |
| Year 12 |  |
| Year 13 |  |

| 5. Have you had a covid vaccine?  (please select an answer) | |
| --- | --- |
| Yes |  |
| No |  |
| Prefer not to say |  |

| 5a. If you have had a covid vaccine, how many covid vaccines have you received? (please select an answer) | |
| --- | --- |
| 1 dose |  |
| 2 doses |  |
| 2 doses and 1 booster |  |
| 2 doses and 2 boosters |  |
| Other (please specify): |  |

| 6. How many people (including you) usually live in your home in a typical week? (please select an answer) | |
| --- | --- |
| 2-3 people |  |
| 4-6 people |  |
| More than 6 people |  |

| 7. Thinking only about when you were at school last week, can you **estimate** how many people you think you came into close contact with? By close contact we mean people less than 2m away (e.g. people sitting around you in class or at lunch), people you spoke to; people you may have had some physical contact with (e.g. shake hands, hug).  (please select an answer) | |
| --- | --- |
| Less than 10 people |  |
| 10-30 people |  |
| 30-50 people |  |
| 50-70 people |  |
| 70-90 people |  |
| More than 90 people |  |
| Other (please specify): |  |

| 8. Now thinking only about last week and when you were NOT at school. This would include evenings and weekends. Can you **estimate** how many people you think you came into close contact with? By close contact we mean people less than 2m away (e.g. people sitting around you in class or at lunch), people you spoke to; people you may have had some physical contact with (e.g. shake hands, hug).  (please select an answer) | |
| --- | --- |
| Less than 10 people |  |
| 10-30 people |  |
| 30-50 people |  |
| 50-70 people |  |
| 70-90 people |  |
| More than 90 people |  |
| Other (please specify): |  |

**Section B**

**In the following section, we are going to ask you some questions about your experiences of wearing face coverings. There are no right or wrong answers to these questions**

| 1. How often over the past week did you wear a face covering while indoors at schools (excluding times you were eating or drinking)?  (please select an answer) | |
| --- | --- |
| Never (0%)  [Skip to Section C] |  |
| Up to 25% of the time (e.g., all day but only on one day or for some mornings or some afternoons but not all)  [continue to questions 1a] |  |
| Between 25% and 50% of the time (e.g., half of the day on most days)  [continue to questions 1a] |  |
| Between 50% and 75% of the time (e.g., at least half of the week)  [continue to questions 1a] |  |
| Between 75% and 100% of time (e.g., almost all week)  [continue to questions 1a] |  |
| Always (100%)  [continue to questions 1a] |  |
| N/A - I’m medically exempt (skip all following questions)  [Skip to Section C] |  |

| 1a. Can you describe which part of the day over the past week was the easiest for you to wear masks (e.g., a particular lesson or a particular time of day)? |
| --- |
| 1b. (not asked if participant responds ‘Never’ or ’Always’ or ‘NA’ to (1)) Optional: Can you describe which part of the day over the past week was the hardest for you to wear masks (e.g., a particular lesson, or a particular time of day)? |

| 2. To what extent has wearing a face covering affected your learning over the past week? (please select an answer) | | | | |
| --- | --- | --- | --- | --- |
| Extremely positively | Somewhat positively | Not much/same as not wearing a face covering | Somewhat negatively | Extremely negatively |
| 2a. Please tell us in a few words how it has affected you: | | | | |

| 3. To what extent has wearing a face covering affected your interactions with your friends over the past week (e.g., found it hard to be understood, had to worry about make-up, etc.)? (please select an answer) | | | | |
| --- | --- | --- | --- | --- |
| Extremely positively | Somewhat positively | Not much/same as not wearing a face covering | Somewhat negatively | Extremely negatively |
| 3a. Please tell us in a few words how it has affected you: | | | | |

| 4. To what extent has wearing a face covering affected your interactions with your teachers over the past week? (please select an answer) | | | | |
| --- | --- | --- | --- | --- |
| Extremely positively | Somewhat positively | Not much/same as not wearing a face covering | Somewhat negatively | Extremely negatively |
| 4a. Please tell us how it has affected you: | | | | |

| 5. Did any of your teachers wear a face mask while teaching you last week? (please select an answer) | | | | | | |
| --- | --- | --- | --- | --- | --- | --- |
| Yes (continue to question 5a) | | | No (skip to question 6) | | Not sure (skip to question 6) | |
| 5a. Did this affect your learning in any way? | | | | | | |
| Extremely positively | Somewhat positively | Not much/Same as if they were not wearing a mask | | Somewhat negatively | | Extremely negatively |
| 5b. Please tell us how it has affected you: | | | | | | |

| 6. To what extent has wearing a face covering affected your interactions with other people in general (e.g., lunch room staff, security staff, etc.) over the past week? (please select an answer) | | | | |
| --- | --- | --- | --- | --- |
| Extremely positively | Somewhat positively | Not much/same as not wearing a face covering | Somewhat negatively | Extremely negatively |
| 6a. Please tell us how it has affected you: | | | | |

| 7. To what extent has wearing a face covering affected your physical health (e.g., headaches, skin problems) over the past week? (please select an answer) | | | | |
| --- | --- | --- | --- | --- |
| Extremely positively | Somewhat positively | Not much/same as not wearing a face covering | Somewhat negatively | Extremely negatively |
| 7a. Please tell us how it has affected you: | | | | |

| 8. To what extent has wearing a face covering affected your mental health over the past week? (please select an answer) | | | | |
| --- | --- | --- | --- | --- |
| Extremely positively | Somewhat positively | Not much/same as not wearing a face covering | Somewhat negatively | Extremely negatively |
| 8a. Please tell us how it has affected you: | | | | |

| 9. During the past week, did you feel uncomfortable being seen wearing a face mask at school? (please select an answer) | | |
| --- | --- | --- |
| Yes | No | Not sure |

**Section C**

**In this section, we are going to ask you about how you have felt during the past week. There are no right or wrong answers to these questions.**

| 1. Please think about what you have been doing and experiencing during the last week. Then report how much you experienced each of the following feelings, using the scale below.  For each item, select an option from "Very rarely or never" to "Very often or always". | | | | | |
| --- | --- | --- | --- | --- | --- |
| **Positive** | Very rarely or never | Rarely | Sometimes | Often | Very often or always |
| **Negative** | Very rarely or never | Rarely | Sometimes | Often | Very often or always |
| **Good** | Very rarely or never | Rarely | Sometimes | Often | Very often or always |
| **Bad** | Very rarely or never | Rarely | Sometimes | Often | Very often or always |
| **Pleasant** | Very rarely or never | Rarely | Sometimes | Often | Very often or always |
| **Unpleasant** | Very rarely or never | Rarely | Sometimes | Often | Very often or always |
| **Happy** | Very rarely or never | Rarely | Sometimes | Often | Very often or always |
| **Sad** | Very rarely or never | Rarely | Sometimes | Often | Very often or always |
| **Afraid** | Very rarely or never | Rarely | Sometimes | Often | Very often or always |
| **Joyful** | Very rarely or never | Rarely | Sometimes | Often | Very often or always |
| **Angry** | Very rarely or never | Rarely | Sometimes | Often | Very often or always |
| **Contented** | Very rarely or never | Rarely | Sometimes | Often | Very often or always |

| 2. Over the past week, how often did you feel that you have no one to talk to? | | | | | | |
| --- | --- | --- | --- | --- | --- | --- |
| Hardly ever or never | | Some of the time | | | Often | |
| 3. Over the past week, how often did you feel left out? | | | | | | |
| Hardly ever or never | | Some of the time | | | Often | |
| 4. Over the past week, how often did you feel alone? | | | | | | |
| Hardly ever or never | | Some of the time | | | Often | |
| 5. Over the past week, how often have you felt lonely? | | | | | | |
| Often/always | Some of the time | | Occasionally | Hardly ever | | Never |

| 6. Is there anything else you’d like to tell us about your experience of being part of the Face It Study so far? |
| --- |

**Section D (This section is to be added to the last survey)**

**In this section, we are going to ask you a few questions about your experience of providing saliva samples.**

| 1. Were the instructions on how to collect your saliva samples easy to understand? | | | | |
| --- | --- | --- | --- | --- |
| Very easy | Easy | Neither easy nor difficult | Difficult | Very difficult |

| 2. How would you describe the process of collecting the saliva samples? | | | | |
| --- | --- | --- | --- | --- |
| Very easy | Easy | Neither easy nor difficult | Difficult | Very difficult |

| 3. Did you experience any discomfort when collecting your saliva samples? | | |
| --- | --- | --- |
| A lot of discomfort | A little discomfort | None |

| 4. Is there any part of the saliva sample collection you would change to make it easier for you to do the test? If so, please explain below |
| --- |

| 5. Is there anything else you’d like to tell us about your experience of being part of the Face it Study so far? |
| --- |

| 6. If you were to receive an incentive to participate (eg an Amazon voucher or similar) would you be more likely to participate? | | | | | |
| --- | --- | --- | --- | --- | --- |
| Yes definitely | Yes possibly | I don’t know | | Possibly not (skip to 7) | No, a voucher wouldn’t affect my decision (skip to 7) |
| 6a. Would you prefer to receive one voucher at the end as a thank you for taking part, or a voucher every week that you participated? | | | | | |
| One at the end | | | A voucher every week | | |

| 7. If you were able to choose where you provided your saliva sample (eg at home, when on your own), would you be more likely to participate? | | | | |
| --- | --- | --- | --- | --- |
| Yes definitely | Yes possibly | I don’t know | Possibly not | No, it wouldn’t affect my decision |

| 8. If you were given reusable masks to wear, would you be more likely to participate? | | | | |
| --- | --- | --- | --- | --- |
| Yes definitely | Yes possibly | I don’t know | Possibly not | No, it wouldn’t affect my decision |

| 9. If we made all the changes you have suggested above in terms of an incentive, saliva samples and face masks, would you be more likely to participate? | | | | |
| --- | --- | --- | --- | --- |
| Yes definitely | Yes possibly | I don’t know | Possibly not | No, it wouldn’t affect my decision |

**You’ve reached the end of this survey. Thank you very much!**

## Trial Procedure and Adjustments

|  | **Survey data collection** | **Saliva sample collection** |
| --- | --- | --- |
| **Baseline (13/09/2020)** | Pupils completed survey through QR codes during the weekly assembly, immediately following Face It presentation. Hard copies of the survey were also available for pupils unwilling or unable to complete the online survey. | Fresh saliva kits were left on seats in the hall. Pupils who took part in the study were encouraged to provide a saliva sample immediately after completion of the online survey. |
| **Week 1 (20/09/2020)** | Same as Baseline | Same as Baseline |
| **Week 2 (27/09/2020)** | Same as Baseline | Same as Baseline |
| **Week 3 (04/10/2020)** | QR code and link to the survey were sent to school a day before. Researchers visited each tutor group at the regular research visit time (i.e., Tuesday morning 8.30am). At each tutor group, participants were encouraged to scan the QR code and complete the survey. | Participants were encouraged to take a fresh saliva kit immediately after completing the online survey. Participants were able to return the saliva sample after tutoring time (i.e., at around 9am). |
| **Week 4 (11/10/2020)** | Same as Week 3 | Fresh saliva kits were dropped off at the school a day before. A teacher distributed the fresh saliva kits to all year 12 and year 13 pupils who took part in the study upon them entering the school (approximately 8am). Pupils were told that they would be able to provide their saliva sample anytime before the regular research visit. Participants were rewarded with a selection of confectionary upon returning saliva samples. |
| **Week 5**  **(18/10/2020)**  *Pupils doing medicine courses were not present at school on Tuesday morning during regular research visit time due to educational/placement arrangements. | QR code to the online survey were printed and left on seats in the hall. A digital copy was also available on the screen in the same room. Pupils were instructed to scan the QR code and complete the survey when they were seated. | Fresh saliva kits were dropped off at the school a day before. A teacher distributed the fresh saliva kits to all year 12 and year 13 pupils who took part in the study upon them entering the school (approximately 8am). Small rewards (confectionary) were given to pupils who took the fresh saliva kit. Pupils were told that they would be able to provide their saliva sample anytime before the regular research visit. Participants were instructed to leave their samples in the collection box while entering the hall and were rewarded with a selection of sweets immediately. |

## Summary of results for end of trial feedback and recommendations for future work

| **Question** | **Feedback** |
| --- | --- |
| Were the instructions on how to collect your saliva samples easy to understand? | Very easy (39%) |
|  | Easy (37%) |
|  | Neither easy nor difficult (22%) |
|  | Difficult (0%) |
|  | Very difficult (2%) |
| How would you describe the process of collecting the saliva samples? | Very easy (23%) |
|  | Easy (23%) |
|  | Neither easy nor difficult (32%) |
|  | Difficult (13%) |
|  | Very difficult (9%) |
| Did you experience any discomfort when collecting your saliva samples? | A lot of discomfort (30%) |
|  | A little discomfort (43%) |
|  | None (30%) |
| If you were to receive an incentive to participate (e.g., an Amazon voucher or similar) would you be more likely to participate? | Yes definitely (47%) |
|  | Yes possibly (23%) |
|  | I don’t know (6%) |
|  | Possibly not (5%) |
|  | No, it wouldn’t affect my decision (19%) |
| Would you prefer to receive one voucher at the end as a thank you for taking part, or a voucher every week that you participated? | One at the end (41%) |
|  | A voucher every week (59%) |
| If you were able to choose where you provided your saliva sample (e.g., at home, when on your own), would you be more likely to participate? | Yes definitely (32%) |
|  | Yes possibly (24%) |
|  | I don’t know (20%) |
|  | Possibly not (9%) |
|  | No, it wouldn’t affect my decision (15%) |
| If you were given reusable masks to wear, would you be more likely to participate? | Yes definitely (9%) |
|  | Yes possibly (21%) |
|  | I don’t know (19%) |
|  | Possibly not (17%) |
|  | No, it wouldn’t affect my decision (34%) |
| If we made all the changes you have suggested above in terms of an incentive, saliva samples and face masks, would you be more likely to participate? | Yes definitely (21%) |
|  | Yes possibly (43%) |
|  | I don’t know (14%) |
|  | Possibly not (5%) |
|  | No, it wouldn’t affect my decision (17%) |

# Supplementary Appendix D

## Table D1. Mood by year groups and weeks

|  | **SR (control)** | **FM (intervention)** |
| --- | --- | --- |
| **Positive mood (mean, SD)** |  |  |
| Baseline | 20.8 (3.9) | 17.8 (4.5) |
| Week 1 | 19.9 (4.4) | 16.9 (5.1) |
| Week 2 | 20.0 (3.8) | 16.3 (4.6) |
| Week 3 | 20.2 (4.3) | 15.0 (5.0) |
| Week 4 | 19.4 (3.8) | 15.7 (5.3) |
| Week 5 | 19.9 (3.5) | 17.9 (5.9) |
| **Negative mood (mean, SD)** | |  |
| Baseline | 14.3 (5.0) | 16.5 (4.9) |
| Week 1 | 14.4 (5.1) | 16.6 (5.1) |
| Week 2 | 13.7 (4.0) | 17.0 (5.1) |
| Week 3 | 13.3 (4.6) | 17.3 (5.2) |
| Week 4 | 15.1 (3.3) | 15.9 (7.0) |
| Week 5 | 14.7 (4.2) | 15.9 (5.3) |
| **Loneliness (mean, SD)** |  |  |
| Baseline | 5.1 (1.5) | 4.8 (1.6) |
| Week 1 | 4.7 (1.7) | 4.9 (1.5) |
| Week 2 | 4.4 (1.4) | 4.4 (1.4) |
| Week 3 | 4.3 (1.6) | 5.8 (1.9) |
| Week 4 | 4.7 (1.4) | 5.1 (2.1) |
| Week 5 | 4.5 (1.6) | 5.0 (2.5) |

*Note. Positive and negative mood using The Scale of Positive and Negative Experience* [40]*. We measured loneliness using the Three-Item Loneliness Scale* [41]*.*
